# The proteome of Hypobaric Induced Hypoxic Lung: Insights from Temporal Proteomic Profiling for Biomarker Discovery

**DOI:** 10.1038/srep10681

**Published:** 2015-05-29

**Authors:** Yasmin Ahmad, Narendra K. Sharma, Mohammad Faiz Ahmad, Manish Sharma, Iti Garg, Mousami Srivastava, Kalpana Bhargava

**Affiliations:** 1 Defence Institute of Physiology & Allied Sciences (DIPAS), DRDO, Ministry of Defence, Timarpur, Delhi, India; 2 School of Biotechnology, JNU, New Delhi, India

## Abstract

Exposure to high altitude induces physiological responses due to hypoxia. Lungs being at the first level to face the alterations in oxygen levels are critical to counter and balance these changes. Studies have been done analysing pulmonary proteome alterations in response to exposure to hypobaric hypoxia. However, such studies have reported the alterations at specific time points and do not reflect the gradual proteomic changes. These studies also identify the various biochemical pathways and responses induced after immediate exposure and the resolution of these effects in challenge to hypobaric hypoxia. In the present study, using 2-DE/MS approach, we attempt to resolve these shortcomings by analysing the proteome alterations in lungs in response to different durations of exposure to hypobaric hypoxia. Our study thus highlights the gradual and dynamic changes in pulmonary proteome following hypobaric hypoxia. For the first time, we also report the possible consideration of SULT1A1, as a biomarker for the diagnosis of high altitude pulmonary edema (HAPE). Higher SULT1A1 levels were observed in rats as well as in humans exposed to high altitude, when compared to sea-level controls. This study can thus form the basis for identifying biomarkers for diagnostic and prognostic purposes in responses to hypobaric hypoxia.

Physiological responses to hypoxia have been appreciated for many years and it is well established that healthy individuals exposed to high altitude hypoxia or a chronic hypoxic condition adopt protective mechanisms such as increased breathing, pulmonary vascular constriction or production of erythropoietin to enhance red blood cell mass^1^,^2^. Tissue hypoxia may result from many conditions including environmental (high altitude exposure) or pathological conditions (obstructive sleep apnea (OSA), acute lung injury, asthma attacks, atelectasis, high altitude pulmonary edema (HAPE) chronic obstructive pulmonary disease, and idiopathic pulmonary hypertension) and can be present as acute or chronic manifestation. However, acute and chronic hypoxic responses vary greatly depending on tissue type.

The lung is a unique tissue compared with many other vital organs since it is directly exposed to high levels of oxygen. One of the most important functions of lungs is to maintain an adequate oxygenation in the organism. This organ can be affected by hypoxia facing both physiological and pathological situations. Exposure to hypoxia results in the increase of reactive oxygen species from mitochondria, as from NADPH oxidase, xanthine oxidase/reductase, and nitric oxide synthase enzymes, as well as establishing an inflammatory process. In lungs, hypoxia also alters the levels of antioxidant substances causing pulmonary oxidative damage. Imbalance of redox state in lungs induced by hypoxia has been suggested as one of the causal factors for the changes in lung function in the hypoxic context, such as hypoxic vasoconstriction^3^,^4^ and pulmonary edema^5^,^6^,^7^ in addition to vascular remodelling^8^ and chronic pulmonary hypertension^9^,^10^,^11^.

Recently, discovery proteomics has emerged as a means of qualitatively differentiating specific proteins within complex samples such as lung or lung lavage fluid. Compared to microarray based analysis of gene expression patterns, which has already been undertaken in hypoxic lung^12^,^13^,^14^, proteomic profiling offers distinct advantages. When applied to hypoxic lung, discovery proteomics not only has the potential to provide insight into the key effectors of lung inflammation/injury and repair, but also uncover mechanisms regulating gene expression. Research involving proteomics methods have been applied to the study of hypoxic lung by several groups of investigators. Henschke and co-workers used an electrophoresis-based proteomic method to elucidate the effects of hyperoxia on protein expression in fetal rabbit lungs and to identify putative response pathways that mediates antioxidant and inflammatory processes^15^. Laudi and colleagues compared the *in vivo* changes in the lung proteome in monocrotaline and hypoxia induced pulmonary hypertension using 2-DE coupled with nanoflow LC-MS/MS to study the differences in the underlying mechanism of the regulation of vasoconstriction and remodelling^16^. Kwapiszewska and co-workers used a shotgun proteomic approach to identify chloride intracellular channel 4, as a novel multifunctional protein involved in angiogenesis and several signalling pathways implicated in pulmonary arterial hypertension^17^. Recently, lung proteome profiles of chronic hypoxic rats with pulmonary hypertension were compared with normoxic rats using 2D-based proteomics approach to identify the novel signalling pathways involved in the pathophysiology of pulmonary hypertension^18^. Most recently, Olmeda *et al.* reported a proteomic approach to describe the changes in protein complement induced by moderate long-term hypoxia (rats exposed to 10% O_2_ for 72 h) in BAL and lung tissue, with a special focus on the protein associated with pulmonary surfactant^19^. These studies applied proteomics techniques to study proteins in lung exposed only to chronic hypoxia at a single time point but they did not address the complex and dynamic changes that occur during the course of hypoxia at different time points.

Thus, the purpose of this study was to use proteomic analysis to profile the proteomic changes in the lungs exposed to short-term temporal (0, 6, 12 and 24 h) hypobaric hypoxia and hence, to identify the pathways that are affected due to hypoxia induced lung inflammation. We used a 2D- based proteomic approach to compare the protein profiles in the lung of rat treated with hypobaric hypoxia at different exposure time points. Several hypoxia-regulated proteins were identified by MALDI TOF/TOF. We then applied advanced methods in computational analysis to map complex protein interactions in the lung exposed to hypobaric hypoxia and studied how these interactions changed during the different exposure time points. This approach to protein network analysis identified novel mediators of acute lung hypoxia which were involved in multiple biological processes. These characteristics of the protein interactions in the lungs of rats treated with hypobaric hypoxia have important implications for the development of new molecular-based therapies.

## Materials and Methods

### Experimental Animals and Hypobaric Hypoxia Exposure

Adult male Sprague Dawley rats (n = 24) weighing approximately 220 ± 10 g were used for the study. Animals were maintained in the animal house facility of the Defence Institute of Physiology and Allied Science (DIPAS) with 12-hr light/dark cycle and were provided with food and water at *ad libitum*. Total 24 rats were divided into four groups. Group I served as normoxia (n = 6) maintained in standard environment. Groups II, III and IV served as hypoxia exposure groups for 6 h, 12 h and 24 h respectively (n = 6), where the rats were exposed to simulated hypobaric hypoxia at 7600 m (25,000 ft, 282 mm Hg) in a specially designed animal decompression chamber (Seven Star, India) where altitude could be maintained by reducing the ambient barometric pressure and temperature and humidity could be precisely controlled. The temperature and humidity was maintained at 28 ± 2 °C and 60 ± 5% respectively. The rate of ascent to altitude was maintained at the rate of 300 m/min and it took a period of 20–25 min to reach the desired altitude. All animal procedures and experimental protocol were approved by Institutional Animal Ethics Committee (Authorization Number: 27/1999/CPCSEA) and followed the standards set forth in the Guide for the Care and Use of Laboratory Animals (National Academy of Science, Washington, D.C.). All efforts were made to minimize the animal suffering and to reduce the number of the animals used. The methods were carried out in accordance with the approved guidelines.

### Sample Preparation

After hypoxic exposure, both control (normoxic) and hypoxia exposed rats were deeply anesthetized with sodium pentobarbital (100 mg/kg, i.p.), perfused transcardially with chilled PBS and decapitated. The lungs were collected and used fresh or stored in liquid nitrogen for further use. For protein isolation, 100 mg (wet weight) of lung was homogenized on ice with a tissue tearer in 500 μl lysis buffer [(40 mM Tris (pH 7.5), 8 M urea, 2.5 M thiourea, 3% 3-[(3-Cholamidopropyl) dimethylammonio]-1-propanesulfonate (CHAPS), 10 mM dithiothreitol (DTT), 1 mM ethylenediamine tetraacetic acid (EDTA), 1 mM phenylmethyl- sulfonyl fluoride (PMSF)], and protease inhibitor cocktail. The homogenate was sonicated, vortexed, and centrifuged at 15,000 × g at 4 °C for 45 min. The supernatant was collected and protein concentration was estimated using Bradford reagent.

### Human Subjects and Sample Collection

Blood plasma of stringently selected HAPE patients (n = 10) was compared with healthy male sea level control (n = 10). The plasma from HAPE patients were collected at high-altitude medical research centre (HAMRC, Leh, India). All patients had their diagnosis confirmed by degree of Acute Mountain Sickness (AMS) that was determined by the Lake Louise score by the same interviewer. AMS was diagnosed when the Lake Louise score was >5. Chest radiogram was performed when the clinical assessment and/or the analysis of blood gas indicated HAPE by X-ray unit (TRS, Siemens) at HAMRC (Leh, India) with a fixed target to film distance of 140 cm at 95 kV and 3–6 mA/s. HAPE was diagnosed if the X-ray film showed interstitial and/or alveolar edema compared with the chest radiograph taken at lower altitude. Equal numbers of healthy male subjects were taken as controls, with no previous history for risk factors. Prior to the present study they had not been exposed to high altitude. The plasma from the healthy sea level controls were collected in Defence Institute of Physiology and Allied Sciences (Delhi, India). Volunteers were provided details of the study design as approved by the Ethical Committee of Defence Institute of Physiology and Allied Sciences and a written consent was obtained. The methods were carried out in accordance with the approved guidelines. The physical characteristics of these two groups are summarized (Supplementary File 1, Table S1). Fasting venous blood samples were collected from an antecubital vein in EDTA – treated vials in the morning (0800 to 0900 AM), at sea level and stored at 4 °C until preparation to prevent coagulation and minimize protein degradation. To prepare plasma, blood samples were centrifuged at 1500 g for 10 mins at 4 °C. Supernatants were transferred to new tubes as aliquots. To each 1.0 mL plasma aliquots, 10 μl of protease inhibitor were added. The plasma samples were stored at −80 °C until the assay. All samples used in this study were prepared within 1 hour of sample collection and showed no signs of hemolysis.

### Two-dimensional Gel Electrophoresis

Isoelectric focusing was performed with Immobiline Dry Strip, pH 4–7, 18 cm (GE Healthcare, Sweden) on IPGphor IEF System (GE Healthcare, Sweden) at constant voltage. The strip was pre-incubated with 350 μl rehydration buffer containing 7 M urea, 2 M thiourea, 1.2% w/v CHAPS, 0.4% w/v ABS-14, 20 mM dithiothreitol (DTT), 0.25% v/v pH 3–10 ampholytes, 0.005% w/v bromophenol blue (BPB) and 200 μg protein at 20 °C for 18 h. The IEF condition contained 500 V for 7 h (slow), 1000 V for 1 h (linear), 8000 V for 3 h (gradient), 8000 V for 3 h (linear), 10000 V for 2 h (gradient) and 10000 V for 1 h (linear), with the total of 65–70 KV h. Prior to the second-dimensional gel separation, the IPG strips were equilibrated for 2 × 15 min with gentle shaking in 6 ml of SDS equilibration buffer [50 mM Tris–Cl (pH 8.8), 6 M urea, 30% v/v glycerol, 2% SDS]. DTT (2%, w/v) was added in the first step and iodoacedamide (2.5%, w/v) in the second equilibration step. The second dimension was carried out using EttanDaltSix Electrophoresis System (GE Healthcare, Sweden). The strips were then loaded onto 12% SDS-polyacrylamide gel and sealed with 0.5% agarose. The running buffer contained 25 mM Tris–HCl, pH 8.3, 192 mM glycine and 0.1% w/v SDS. Electrophoresis was performed at a constant current of 25 mA per gel at 25 °C for 6 h. After electrophoresis, proteins were visualized by modified silver staining procedure compatible with MS^20^. The gels were fixed in 50% v/v methanol, 12% v/v acetic acid and 0.05% v/v formaldehyde for at least 2 h. The fixed gels were rinsed with 50% v/v ethanol three times for 20 min each, then again sensitized with 0.02% w/v sodium thiosulfate followed by three washings with milli-Q water each for 20 s. The gels were immersed in 0.1% w/v silver nitrate and 0.075% v/v formaldehyde for 20 min and rinsed with milli-Q water twice for 20 s each. It was developed with 6% sodium carbonate and 0.05% v/v formaldehyde. Finally, the reaction was terminated by fixing with 50% v/v methanol and 12% v/v acetic acid.

### Image Acquisition and Data Analysis

The stained gel images were captured using an Investigator™ ProPic II (Genomics Solutions, UK) and the digitized gel images analyzed using 2D-Progenesis Samespot Software (Non Linear Dynamics, USA). Spot matching between all the gels were viewed using the automatic spot detection and normalization tool and edited where appropriate. The relative intensity of individual spots in 2-DE gels of different time exposure (6, 12 and 24 h) and control rats were quantified using a gray scale and the differences between spot pairs were determined. The median differences of relative spot intensities in matched spots were calculated. For each group, there were six biological replicates and each replicate were analysed twice by 2D analysis.

### MS Identification of Proteins

#### In-gel digestion with Trypsin and Extraction of Peptides

The procedure for in-gel digestion of protein spots from silver stained gels was performed according to Shevchenko *et al.* protocol^21^. In brief, protein spots were extensively washed with ultrapure water and each gel spot was excised with a clean scalpel. The spots were destained and incubated for 30 min with 30 mM Potassium ferricyanide and 100 mM sodium thiosulfate at room temperature. The gel pieces were rinsed several times with water to remove destaining solution. The gel pieces were washed for 15 min at room temperature with water and 50 mM NH_4_HCO_3_/Acetonitrile. Enough acetonitrile were added to cover gel pieces for shrinking the gel pieces. The gel pieces were rehydrated in 10 mM NH_4_HCO_3_ for 5 min, equal volume of acetonitrile were added and removed after 15 min of incubation. The gel pieces were again covered with acetonitrile and removed. The gel pieces were dried in a vacuum centrifuge. The dried gel pieces were digested with 20 ul of trypsin (20 ng/ul, Trypsin Singles^TM^ Proteomics Grade, Sigma) at 37 °C overnight. The trypsin digested peptides were sonicated for 10 min and dried in a speed Vac. The dried peptides were extracted with 5 ul of 0.1% TFA.

### Mass Spectrometry and Database Searching

For PMF, in-gel tryptic peptides of each spot of interest were mixed with an acidic solid matrix such as α-cyano-4-hydroxy cinnamic acid (CHCA, Bruker Daltonics) matrix 10 mg/ml, which provides high sensitivity and negligible matrix adduction during the laser absorption and subjected to laser radiation. The matrix was made in 70% acetonitrile and 0.03% TFA. 0.5 μl of the peptide extracts mixed with the 0.5 μl of the matrix were manually spotted onto a 600 μm/384 well AnchorChip^TM^ sample target (Bruker Daltonics) and dried at ambient temperature. Peptide mass spectra were recorded in the reflectron mode using an Ultraflex III TOF/TOF mass spectrometer (Bruker Daltonics) equipped with a 384-sample scout source. The ion acceleration voltage after pulsed extraction was 29000 V. A peptide calibration standard (Bruker Daltonics) was used for external calibration as previously described^22^. MS and MS/MS data were recorded automatically on the MALDI-TOF/TOF instrument using the three most abundant peptide signals of the corresponding peptide mass fingerprint (PMF) spectrum. The monoisotopic peak list was generated in Post Processing s/w and True peptide mass list was generated by Bruker Flex Analysis software version 3.0 and Biotools ver 3.1 without using the smoothing function and the peak filter was applied to exclude the masses lower than 700 Da and the signal to noise ratio of 20. The generated peptide mass list was searched with MASCOT (http://www.matrixscience.com) using entire Uniprot/Swiss-Prot protein database to find and match the protein identity. Databases searches were performed by using following search parameters; *Rattus norvegicus* as taxonomy, carbamidomethyl modification of cysteines and possible oxidation of methionine, one missed cleavage, a mass accuracy of ≤100 ppm was requested for PMF and for MS/MS searches, a mass accuracy of ≤70 ppm was allowed for peptide masses and their fragments, respectively. For each identified Protein, at least one Peptide was selected for MS/MS (TOF/TOF) to validate the Protein Identity. Instrument was used in the Lift mode (TOF/TOF) to obtain the MS/MS spectra. Again the Flex Analysis 3.0 and Biotools 3.1 s/w were used to generate the fragments mass list and the sequence tag of peptide. The mass list was sent to database in same way as was done in case of above PMF approach. The mass tolerance error of 0.5 Da to 1.0 Da was used for MS/MS ion search. The MS/MS ion search confirmed the protein identity and provided the amino acid sequence of particular peptide. Gene ontology (GO) annotations (functional distribution) for identified proteins were assigned using Blast2GO research tool^23^.

All chemicals were of reagent or electrophoresis grade and, unless specified above, were purchased from GE Health Care and Sigma Chemical Company (St Louis, MO, USA).

### Quantitative Validation by Western Blot Analysis

The protein quantification of 3-hydroxyanthranilate 3, 4-dioxygenase (Haao, Cat. No: ab106436, Abcam, Cambridge, USA), Serum albumin (Alb, Cat. No: B2901, Sigma Aldrich, St Louis, MO, USA), Superoxide dismutase (Sod1, Abcam, Cambridge, USA), Cytochrome b5a (Cyb5a, Abcam-, Cambridge, USA), Regucalcin (Rgn, Abcam, Cambridge, USA), Heat shock protein beta-1 (Hsb1, Cat No: 3093-100, Biovision Incorporated, Milpitas, USA) and 14-3-3 protein zeta/delta (Ywhaz, Cat. No: CG1775, Cell Application Inc, San Diego, USA) were selected to be validated by Western-blot analysis because the expression changes of these proteins were more obvious than that of the other proteins and obtaining their antibodies were convenient. Briefly, whole lung was homogenized in RIPA buffer containing protease inhibitor cocktail. The suspension was centrifuged at 13,000g for 20 min at 4 °C. The supernatant was collected and protein concentration was determined by the Bradford method. Protein (20–40 μg) from each sample was separated on 10–12% SDS-polyacrylamide gel and transferred onto nitrocellulose membranes. Nonspecific binding of protein was blocked by saturating the nitrocellulose membranes with 5% BSA overnight at 4 °C. The membranes were rinsed with PBST and incubated with primary antibodies for 2 h, followed by secondary antibody for another hour. All these experiments were conducted at room temperature. The immunocomplexes were visualized by chemiluminescence using the chemiluminescent peroxidase substrate kit (Sigma-Aldrich, St. Louis Mo 63103, USA). Autoradiogram signals were captured using a gel documentation system (Model Omega, Ultra Lum Inc., USA) and quantitative densitometry analysis was done with ImageJ software (Version 1.6, National Institutes of Health, MD). The quantitative data were normalized against β-actin and the data are expressed as percentage of mean control values (n = 3).

### Semiquantitative RT-PCR

Total RNAs of rat lungs were isolated by RNeasy Mini Kit (Qiagen Inc., Valencia, CA, USA) according to the manufacturer’s instructions. The quantity of total RNA isolated from each sample was quantified by measuring absorbance at 260 nm and the purity was assessed by the A_260/280_ ratio. Samples with A_260/280_ ratio between 1.8 and 2.0 were taken for further proceedings. Total isolated RNA was reverse transcribed using Enhanced AMV Reverse Transcriptase Enzyme system (Sigma, St Louis, MO) according to the manufacturer’s protocol. Briefly, total RNA (1 μg) was mixed with 500 μM dNTPs, 2.5 μM random nonamers and the final volume was made up to 10 μl with water. The tube containing the reaction mixture was incubated at 70 °C for 10 min and subsequently at 25 °C for 15 min. 10 μl of reverse transcriptase reaction mixture containing 2 μl of 10X AMV-RT reaction buffer; 1 μl of RNase inhibitor (1 U μl^−1^); 1 μl of Enhanced avian RT (1 U μl^−1^) and 6 μl of water was added to each sample tube and incubated at 42 °C for 50 min. The cDNA solution was then stored at −20 °C and used as the template for amplification in the PCR. Semiquantitative RT-PCR analysis was employed to validate the differential expression of proteins Haao, Alb, Sod1, Cyb5a, Rgn, Hspb1, Sult1a1 and 14-3-3 protein zeta/delta in rat lungs. The primers of these proteins were designed using the primer premier 5.0 software, and their specificity was tested by SequencherTM software to avoid amplification of any other related gene members. The ubiquitous housekeeping gene actin was used as the loading control. The sequences of the primers for eight genes are listed in Table 1. PCRs were performed using *TaKaRa Taq* enzyme in a final volume of 25 μL as described by the manufacturer. PCR products were separated by electrophoresis on a 1.5% agrose gel stained with ethidium bromide (EtBr) to enable DNA visualization under UV light. Intensity of bands on agarose gel was quantified using Quantity One Software (Bio-Rad, Gel Doc System).

### Immunohistochemistry

We further selected two proteins (3-hydroxyanthranilate 3, 4-dioxygenase, Haao and Cytochrome b5a, Cyb5a) on the basis of their expression level and physiological roles in hypoxia for validation. Lungs form all the experimental groups were perfused, fixed, removed and further fixed in 10% formaldehyde. Briefly, Lung sections (5 micrometers) were cut from archival paraffm blocks. Sections were heated to 80 °C for 30 min and then deparaffinized in xylene and rehydrated through graded alcohols to distilled water. Sections were then placed in a 10 mM citric acid solution (pH 6.0) and microwaved for two 3-min cycles. Sections were cooled to room temperature and then transferred to PBS for 10 mm. Endogenous peroxidase activity was eliminated by placing sections in 3% hydrogen peroxide for 20 mm. Sections were rinsed in PBS for 10 mm and then blocked in 5% horse serum in TBSTM for 30 mm^24^. Sections were then incubated overnight at 4˚C with primary antibodies against Haao and Cyb5a, in a humidified chamber on shaker. The sections were subsequently washed twice with TBS and incubated with the corresponding secondary antibodies conjugated with alkaline phosphatase. The sections were visualized with 3,3-diaminobenzidine tetrahydrochloride (DAB) and counterstained with hematoxylin. Photomicrographs were obtained with Olympus BX51 microscope at 200X magnification. Computer assisted immunohistochemistry quantification was done using Adobe^®^Photoshop^®^ (version 7.0; Adobe Systems, San Jose, CA) as described by Hans-Anton Lehr^25^. The colour contrast of the original image was increased using hue saturation tools in the image setting menu. Using the Magic Wand tool and the select similar command the DAB stained area (brown chromogen) was separated and pixel count of the chromogen was quantified using the histogram command in the image menu. The values were represented as fraction of chromogen pixels relative to counter stain hematoxylin in selected field area. Every sixth coronal section was analyzed and values were plotted as the percentage change from mean of all the six sections.

### Quantification of Plasma Levels of Sulfotransferase 1A1 as Biomarker in HAPE

To determine the correlation of sulfotranseferase 1A1 levels with HAPE, 20 plasma samples, including 10 HAPE patients and ten from normal control groups, were used for quantitative validation. The total sulfotransferase was quantified using competitive ELISA kit with the purified polyclonal antibody against sulfotransferase 1A1 (CUSABIO, China) according to the manufacturer’s instructions. Briefly, the plasma samples were diluted with sample diluent (1:25 dilutions for sulfotransferase 1A1). 100 μL of the diluted plasma samples were added onto 96- well plate and incubated at 37 °C for 2 h. A 100 μL of biotinylated antibody was added, mixed and incubated for 1 h at 37 °C. After three washes with wash buffer, 100 μL of HRP–avidin (1x) was added and incubated for 1 h at 37 °C. After five washes, 90 μL of TMB substrate was added and incubated for 15–30 min at 37 °C. 50 μL of the stop solution was added to each well and absorbance was measured on a microplate reader at a wavelength of 450 nm immediately.

### Determination of Protein Carbonyl Content

Protein oxidation is the covalent modification of a protein induced either directly by reactive oxygen and nitrogen species (RONS) or indirectly by their reaction with secondary by-products. Redox cycling cations such as Fe^2+^ and Cu^2+^ can bind to cation binding locations in proteins and with the aid of further attack by free radicals can transform side-chain amine groups of several amino acids into carbonyls. These carbonyl derivatives are chemically stable and serve as markers of oxidative stress. Carbonyl content in lung tissues was determined using the Protein Carbonyl Assay Kit (Cayman Chemicals, USA) according to the manufacturer’s instructions. In brief, protein carbonyls present in nucleic acid free tissue homogenate were derivatized to DNP hydrazone, analyzed spectrophotometrically and expressed as nanomoles per milligram of protein.

### Bioinformatics and Functional Analysis

The networks, functional analyses, and canonical pathways were generated through the use of Ingenuity Pathways Analysis (Ingenuity®Systems, http://www.ingenuity.com) and toppgene suite (http://toppgene.cchmc.org). After obtaining the list of proteins which were differentially expressed after hypobaric hypoxia from the MALDI-TOF/TOF analysis, a corresponding gene list was created from this protein list. The list was then analyzed by Toppgene suite to identify the most significant biological functions. Toppgene utilize mouse phenotype data explicitly in their prioritization approaches^26^. A p value less than p ≤ 0.05 were considered significant.

### Network Construction and Pathway Analysis

Protein interaction networks reflecting temporal changes in the lung protein expression after the hypoxia exposure were constructed using the Ingenuity Pathways Analysis software and database (IPA; Ingenuity Systems; Redwood City, CA)^27^. IPA was used to interpret the differentially expressed proteins in terms of an interaction network and predominant canonical pathways. The Ingenuity Pathways Knowledge Base (IKB) is a regularly updated curated database that consists of interactions between different proteins culled from scientific literature. IPA uses this database to construct protein interaction clusters that involve direct and indirect interactions, physical binding interactions, enzyme-substrate relationships, and cis-trans relationships in transcriptional control. The networks are displayed graphically as nodes (proteins) and edges (the biological relationship between the proteins). All edges are supported by at least 1 reference from the literature or from canonical information stored in the Ingenuity Pathways Knowledge Base. The intensity of the node color indicates the degree of up- (red) or down- (green) regulation. Nodes are displayed using various shapes that represent the functional class of the gene product. A protein interaction network was generated as follows. A dataset containing the upregulated proteins, called the focus proteins, for a particular cell line was uploaded into the IPA. These focus proteins were overlaid onto a global molecular network developed from the information in the IKB. Networks of these focus proteins were then algorithmically generated by including as many focus proteins as possible and other non-focus proteins from the IKB that are needed to generate the network based on connectivity. Enriched canonical pathways were identified from the IPA library using a Fisher’s exact test adjusted for multiple hypotheses testing using the Benjamini- Hochberg correction^28^. Several protein hubs that had not been identified in the proteomics experiment but were critical in linking the detected proteins together were identified by the program and incorporated into the network.

### Statistical Analysis

Statistical analyses of proteomic data were performed automatically by the Progenesis Same Spots software (Nonlinear Dynamics, USA). The results of biochemical estimation and immunoblots are representations of three separate experiments (Mean ± SD). Statistical analysis of biochemical and immunoblots were done using GraphPad Prism (Version 5) one-way ANOVA with pair-wise multiple comparison procedures (Student–Newman–Keuls method), and p-value of <0.05 was considered significant. The results of semiquatitative RT-PCR were analyzed by one-way ANOVA, followed by Dunnett’s multiple comparison tests. Statistical analysis of ELISA was done using GraphPad Prism (Version 5) two tailed test and p-value of <0.05 was considered significant.

### PCA

Principal components analysis (PCA) is an exploratory analytical tool that is used to reduce the complexity of the dataset and, also to identify meaningful groups and associations in the dataset. PCA transforms a number of correlated variables (e.g., individual protein spot abundance levels in each experimental sample) into a smaller number of uncorrelated variables, called principal components. The first principal component accounts for as much of the variability in the data as possible, and each succeeding component accounts for successively decreasing amounts of the remaining variability. PCA was used in this study to cluster the experimental groups based on the expression of protein spots in the lung. All protein spots that were present in more than 50% of the gels and identified by mass spectrometry were included in the PCA analysis, which was performed using R-programming package “FactoMineR”^29^. In the resulting graph, the spot maps were plotted in two-dimensional space, showing the principal components PC1 and PC2 that divided the samples analyzed orthogonally according to the two principal sources of variation in the data set.

## Results

### Proteome Changes in Rat Lung during Hypobaric Hypoxia Exposure

**Figure 1:**
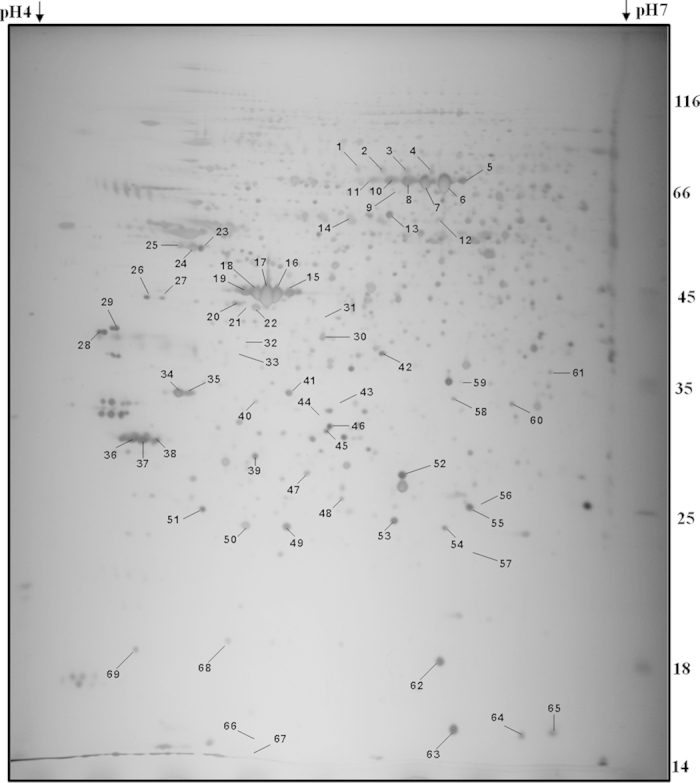
Representative 2D gel image of proteins altered acutely in hypoxic rat lung. Total proteins (200 μg) of each time point were subjected to a 2D-E system (First dimension, IPG strip, pH 4-7NL, 18 cm; Second dimension 12% SDS-PAGE). Proteins were visualized by silver nitrate staining, and 43 proteins were found to vary in a statistically significant way in the hypobaric hypoxia rat model.

The difference between lung proteome profiles of hypoxia treated groups at different exposure times (6, 12 and 24 h) and normoxia control (0 h) was examined using 2-DE with nonlinear IPG ranging from pH 4-7 (Supplementary File 2-5, Figures S2-S5). A total of six lung samples were analyzed in duplicates for each time point and analyzed by 2D-Progenesis Samespot Software and reproducibility (>85%) was achieved. More than 1000 spots were detected on each gel with molecular weights ranging from 14 to 150 kDa and pI values between 4 and 7. Figure 1 shows the master gels of the four groups chosen as reference gels because of their high resolution and large number of protein spots. In the present study, we considered a ≥20% change as significant and sufficient to include all the proteins for which even a 20% change in their expression levels (even at low fold change) is likely to have a functional relevance. The comparison of temporal hypoxic lung proteome with normoxic lung proteome enabled us in the identification of 40 differentially expressed proteins out of 69 spots. The positions of these 40 differentially expressed protein spots were marked with numbers in one representative gel shown in Fig. 1. We were able to detect 26 (20 up- and 6 down-regulated), 30 (26 up- and 4 down-regulated) and 37 (20 up- and 17 down-regulated) proteins with altered levels after 6, 12 and 24 h of exposure, respectively. These 40 significantly deregulated spots were successfully identified by analysis of MALDI-TOF/TOF with PMF and MS/MS followed by database searching (Fig. 1, Table 2). Table 2 lists the proteins identified from these spots with their accession numbers, protein name, Mascot score, theoretical and experimental molecular weights, pIs, sequence coverage, peptide matched, gene name, protein identification and number of folds of protein expression in the four groups. For spots 4, 14, 24, 25, 28, 41–43, 45, 46, 48–50, 53–60 and 64–68, corresponding proteins in the database could not be found even after using the PMF and MS/MS searching. These may be novel proteins or else they may be small fragments of some proteins as can be said based on their molecular weight (≥15 kDa). If they would have been only smaller fragments, the PMF information for these two spots could be limited resulting in the failure of detection of the corresponding proteins in the database. Among these identified proteins, three were detected for the first time in lung samples: regucalcin (Rgn, 8.7 fold), 3-hydroxyanthranilate 3, 4-dioxygenase (Haao, 10.2 fold) and sulfotransferase 1A1 (Sult1a1, 5.2 fold) which were highly up-regulated in lung after 24 h of hypobaric hypoxia exposure as compared to normoxia lung.

### GO -based Analysis of the Identified Proteins

**Figure 2:**
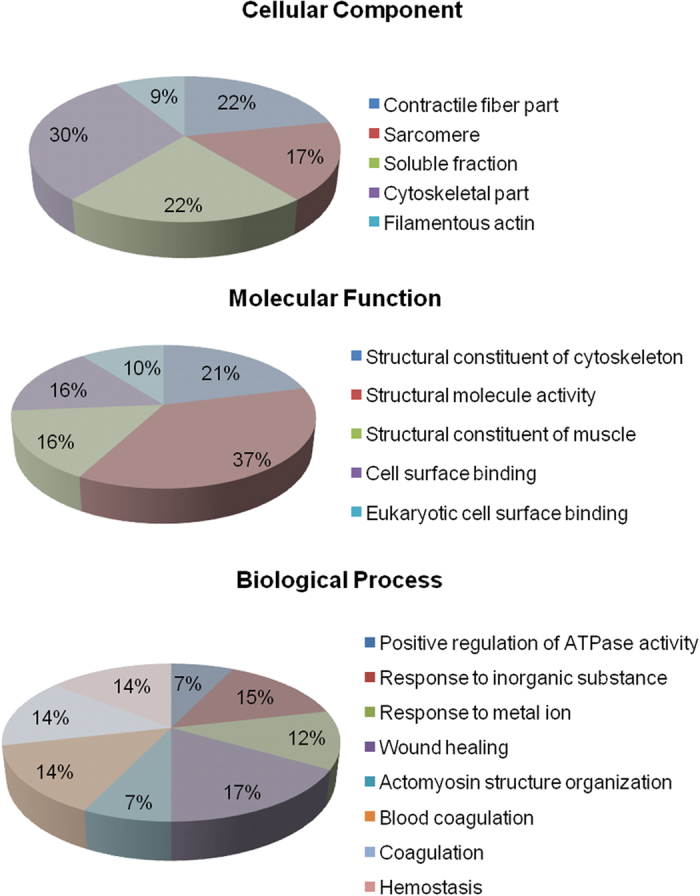
Gene ontology annotations of the proteins identified by MALDI-TOF/MS. Results were obtained using Blast2GO annotation. The distribution of identified proteins according to their (**A**) molecular functions and (**B**) biological processes.

To better characterize the functional differences between proteins detected in the hypoxic lung versus normal controls, the 43 proteins whose expression changed after the hypobaric hypoxia exposure were analyzed using the Gene Ontology (GO) database. The differentially expressed proteins listed here represent a wide range of biological categories. Proteins related to cellular defense mechanisms involving anti-inflammatory and antioxidant activity were the most common. When organized according to their molecular functions, 37% of the identified proteins correspond to those involved in structural molecule activity, 21% involved in structural constituent of cytoskeleton and 16% structural constituent of muscle and cell surface binding activity (Fig. 2). We also categorized the proteins according to their biological processes; most abundant groups of proteins corresponds to those involved in wound healing (17%), positive response to inorganic substance (15%), haemostasis (14%), coagulation (14%), positive regulation of ATPase activity and actomyosin structure organization (7%), with significant p-values. These biological processes are all consistent with changes made in response to hypobaric hypoxia. These classifications provide clues to understand better the proteomic expression changes in the lung during early response to hypobaric hypoxia.

### Pathway Analysis and Protein Interaction Network Generation using IPA

**Figure 3:**
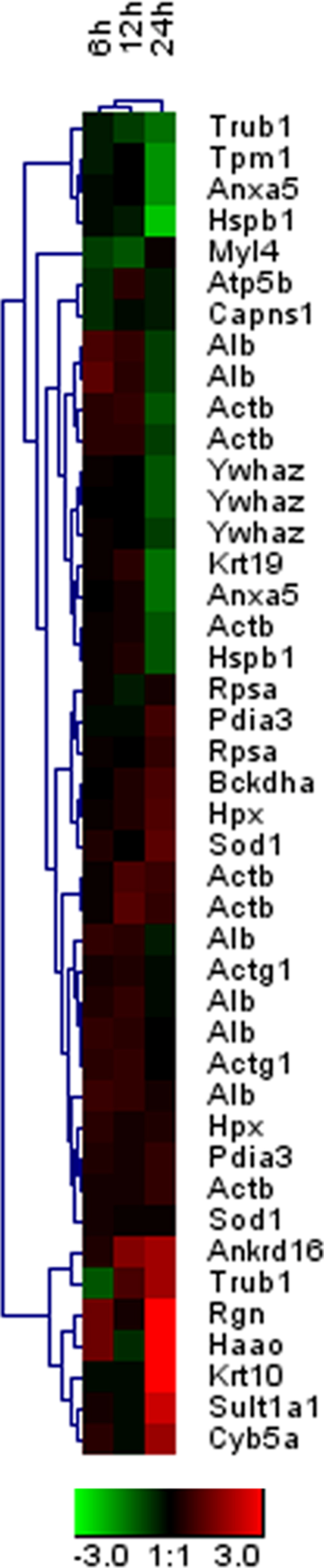
Hierarchical clustering using the differentially expressed proteins in hypoxic rat lungs at different time points. Changes in the expression of the proteins that were identified in the proteomic analysis are shown in a red to green color scale corresponding to the relative abundance compared with the control noromoxic lung (red = up; green = down).

To gain an understanding of functional cellular pathways and processes that may be temporally regulated during exposure to hypobaric hypoxia, we uploaded all the identified significant proteins for lung to the IPA. The top significant functions detected for all the exposure time points for lung are haematological disease and immunological disease (Supplementary File 6, Table S2), Functions like cellular assembly and organization were detected only after 6 h of exposure. It is important to note that an effect like inflammatory disease was detected only after 12 h of hypoxic exposure in lung. We next visualized the molecular networks based on significant functions at each time point for lung using IPA. The networks assembled with IPA profiling of proteins differentially expressed after 6, 12 and 24 h of hypoxia exposure, including interacting partners were depicted for lung. The class functions of each of the proteins are depicted by nodal shape with interaction levels depicted by lines between the nodal shapes. The connectivity among these identified proteins was enhanced by systematically incorporating proteins that have known interactions with the identified proteins but were not detected in the proteomic analysis. The computational network analysis in this study was an attempt to capture the complex and dynamic nature of the changes in the hypobaric hypoxia exposed lungs. The structure of the network that was generated from the proteomic analysis highlighted the complex interactions among its various nodes and identified several proteins as central hubs of connectivity. There is increasing evidence that the functional stability of biological networks is critically dependent on such hubs^30^. In the hypoxic lung protein network, many of the central hubs were proteins that have been studied previously in the pathogenesis of hypobaric hypoxia induced lung inflammation/injury, including NFkB, ERK1/2, JNK and P38 MAPK^31^,^32^. Proteins like PDIA3, ATP5B, ACTIN, ACTG and ACTB occupied the central nodes of networks for 6 and 12 h of exposure while for 24 h of exposure, molecules like YWHAZ, HSPB1, ACTB and SOD occupied central node positions of detected networks. Other highly connected nodes, however, were proteins whose roles have not been well studied in hypoxic lung, including regucalcin, annexin A5, and Keratin, type I cytoskeletal 19. The temporally dynamic characteristic of hypoxic lung was revealed by the time point exposure network analysis. Significant differences were seen in the expression of many members of the network between 6, 12 and 24 h of hypoxia exposure (Supplementary File 7, Figure S6). Protein disulfide-isomerase A3, actin cytoplasmic 2, actin cytoplasmic 1, superoxide dismutase [Cu-Zn], serum albumin, hemopexin and cytochrome b5 were increased in the lung after 6 h exposure compared with normal lung. Conversely, several proteins, including ATP synthase subunit beta, probable tRNA pseudouridine synthase 1, tropomyosin alpha-1 chain, calpain small subunit 1 were decreased after 6 h of hypoxia exposure. The changes in the expression of these proteins in the hypoxic lung likely reflected perturbations in signal transduction, structural molecule activity, proton-transporting ATP synthase activity and apoptotic pathways, as well as cell and oxidative stress during the early phase of hypoxia exposure. In contrast to the significant changes in protein expression between the normal and 6 h of hypoxic lung, the differences between 6 and 12 hr were less dramatic (Supplementary File 7, Figure S6). Nevertheless, many proteins changed in abundance between these times. Some proteins, such as 40S ribosomal protein SA, heat shock protein beta-1 and keratin, type I cytoskeletal 19 showed major differences, while others, including protein disulfide-isomerase A3, hemopexin, actin cytoplasmic 2, actin cytoplasmic 1, serum albumin, protein disulfide-isomerase A3 and actin had modest, but detectable, changes. Some proteins like superoxide dismutase and cytochrome b5 were undetectable in 12 h hypoxic lung exposure. These changes reflect the likely changes in the oxidative stress pathway during the early response to hypobaric hypoxia. The expression profile of the network for 12 h versus 24 h of hypoxic lung also revealed several proteins that changed significantly in expression (Supplementary File 7, Figure S6). These included annexin A5 (decreased), 14-3-3 protein zeta/delta (decreased), keratin type I cytoskeletal 10 (increased), keratin, type I cytoskeletal 19 (decreased), among others. These changes likely reflect regeneration of the lung epithelium, decreased cellular inflammation and resolution of lung inflammation. We have further identified the canonical pathways for hypoxia exposure for lung (Supplementary File 8, Figure S7). Temporal analysis of canonical pathways for lung revealed that acute phase response signalling pathway, VEGF signaling mitochondrial dysfunction and NRF-2 mediated oxidative stress response pathway were identified for all exposure durations suggesting the protective response in defense in hypobaric hypoxia. Interestingly, 14-3-3 mediated signaling was identified in all the exposure points. Similarly, superoxide-radicals degradation pathway, GAP junction signaling and antioxidant action of Vitamin C were identified only for 6 and 12 h of hypoxia exposure (Table 3). Information obtained from IPA analysis relates canonical pathways to a group of genes, but lacks the ability to predict how a pathway is regulated differently. For instance, mitochondrial dysfunction was a significant pathway in all exposure durations; it remained unclear if this pathway was up- or down-regulated. NF-κB complex formed a major hub at the centre of the network in lung hypoxia, with a number of direct and indirect interactions with the focus molecules in the network. Validation of these nodes is an important step as they are major gene regulators and deletion of any of these nodes may influence the inferred network. The changes in protein expression after acute hypobaric hypoxia at early time points (6h, 12h and 24h) compared with normal control (0 h) are summarized in Fig. 3 (heat map).

### PCA

Multivariate statistical analysis by PCA was used to reduce complexity, to discover unique patterns and trends in lung protein expression between the hypobaric hypoxia exposed groups and controls. These samples were grouped according to the variance of their protein expression (%V) and their spatial distribution is shown in Supplementary File 9 Figure S8). The first principal component (PC1) explained 85.84% of the variance and the second (PC2) explained a further 11.91%. As shown in Figure S8, PCA was not able to discriminate control, 6 h exposure and 12 h exposure groupings from one another. In contrast, exposure to hypobaric hypoxia for 24 hours led to dramatic alterations in the protein expression, thereby defining a critical temporal threshold for hypobaric hypoxia –induced alterations to the lung proteome.

### Validation of Proteomics Results using Immunoblotting

**Figure 4:**
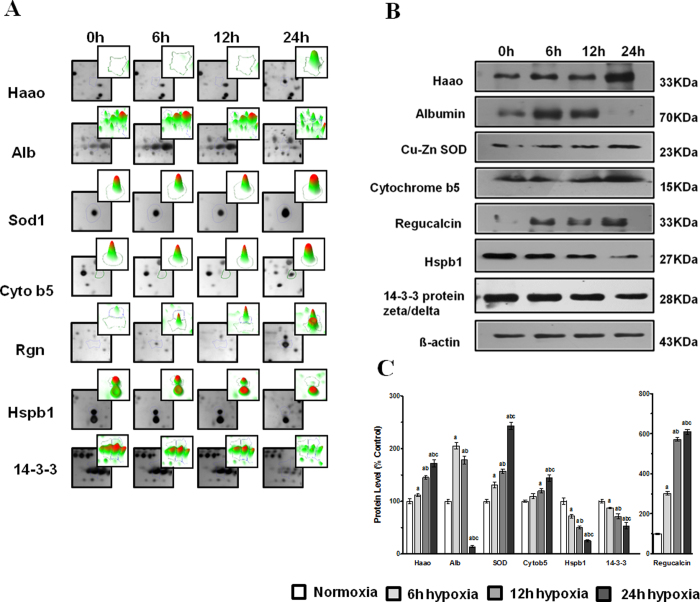
(**A**) Validation of protein change patterns at different time points (0 h, 6 h, 12 h and 24 h) by western blot analysis of proteins Haao, Alb, Sod1, Cyb5a, Rgn, Hspb1 and Ywhaz. (**B**) Total protein (20–40 μg/lane) were separated by SDS-PAGE and probed with the primary antibodies of these seven proteins. (**C**) Densitometric analysis of immunoblots in percentage change with respect to control and signals were normalized against β-actin (Image J, USA). Each bar represents Mean ± SD. ‘a’ refers as P ≤ 0.05 with compare to normoxia, ‘b’ refers as P ≤ 0.05 with compare to 6 h hypoxia and ‘c’ refers as P ≤ 0.05 with compare to 12 h hypoxia.

We verified whether the expression patterns of selected proteins Haao, Alb, Sod1, Cyb5a, Rgn, Hsb1 and Ywhaz observed in 2-DE gels (Fig. 4) paralleled those validated by Western blot analysis. Band intensity was measured with Quantity One 1-D Analysis Software version 4.6.7 BIO-RAD, and the intensity ratio to corresponding β-actin band was calculated. Hsb1 and Ywhaz both were down-regulated in lung after acute hypobaric hypoxia (Fig. 4). The protein levels of Alb increased gradually from 6 to 12 hr but nearly undetectable at 24 hr after acute hypobaric hypoxia. The expression pattern of Sod1 increased gradually from 6 to 24 hr after acute hypoxia (Fig. 4). The expression of Haao increased gradually from 6 to 12 hr but up-regulated about 50 times at 24 hr and Cyb5a were up-regulated in lung after acute hypobaric hypoxia and remained at high levels (Fig. 4). The protein levels of Rgn increased at 6 hr and decreased slightly at 12 hr after acute hypobaric hypoxia but increased significantly at 24 hr exposure as compared to 0 hr (control) where the expression of Rgn were nearly undetectable (Fig. 4). The expression patterns of the selected proteins were in agreement with 2-DE results, so the results of Western blot analysis confirmed the reliability of the proteomic analysis.

### Haao and Cyb5a Immunohistochemistry

**Figure 5:**
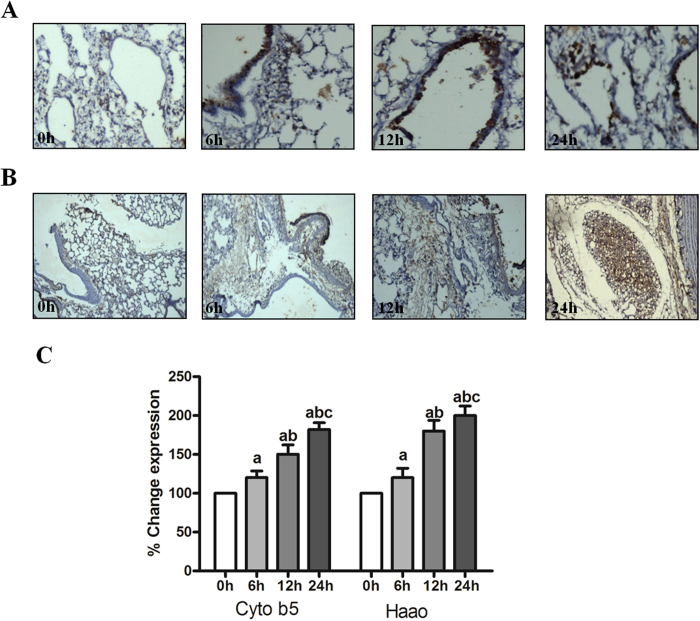
Immunohistochemical analysis of Haao and Cyb5a in rat lungs. (**A**) Immunostaining with an antibody to Haao of normoxic lung (0 h) compared with lung from hypoxic rats treated at different time points (6 h, 12 h and 24 h). Similarly, (**B**) immunostaining with an antibody to Cyb5a of normoxic lung (0 h) compared with hypoxic lung of rats treated at different time points (6 h, 12 h and 24 h). The results showed no positive immunoreactivity for Haao and Cyb5a in control rats (0 h) but Haao and Cyb5a stained strongly in hypoxic lung sections (6 h, 12 h and 24 h). Figure 5 (**C**) showed significantly higher levels of Haao and Cyb5a in hypoxia exposed lung tissues. Data were represented as Mean ± SD of three individual experiments. ‘a’ refers as P ≤ 0.05 with compare to normoxia, ‘b’ refers as P ≤ 0.05 with compare to 6 h hypoxia and ‘c’ refers as P ≤ 0.05 with compare to 12 h hypoxia.

As seen in Fig. 5 under the staining condition used, acute hypobaric hypoxia leads to gradual increase in Haao expression as evident from intensity of spots in gel. Results showed that acute exposure of hypobaric hypoxia for 6 h increases Haao expression but decreases at 12 h exposure. On the other hand maximum changes in expression of Haao were seen after 24 h of acute hypobaric hypoxia exposure. Similarly, Cyb5a level increases gradually from 6 to 12 h of exposure to hypobaric hypoxia and maximum expression were seen after 24 h of hypobaric hypoxia exposure, again the results of immunohistochemistry confirmed the reliability of the proteomic analysis.

### Quantification of Protein Carbonyl Levels

**Figure 6:**
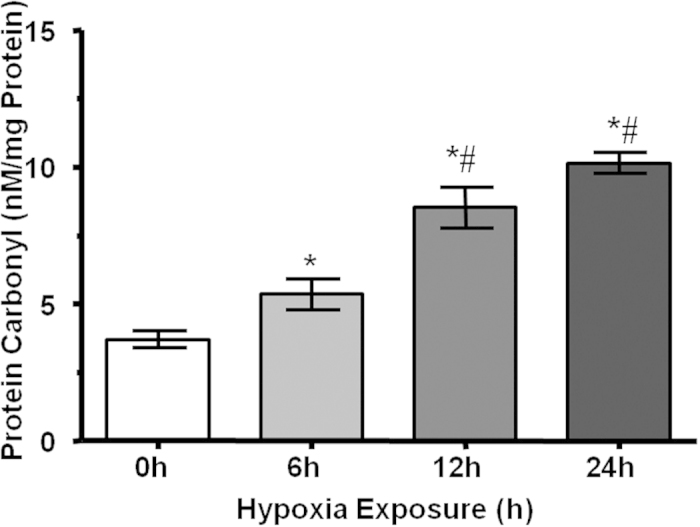
Estimation of protein carbonyl contents in lung tissue after 6 h, 12 h and 24 h of hypobaric hypoxia exposure. The data is expressed as Mean ± SD of three individual experiments. *p < 0.05 when compare with normoxia and #p < 0.05 when compared to corresponding hypoxia group.

We found that the level of protein carbonyls increased with hypoxia duration and highest levels were observed after 24 h of acute hypobaric hypoxia exposure (Fig. 6). The protein carbonyl content of lung was 3.7, 5.4, 8.5 and 10.13 nM/mg protein corresponding to 3, 6, 12 and 24 h of hypoxic stress respectively.

### Validation of the Expression Pattern using Semiquantitative RT-PCR

**Figure 7:**
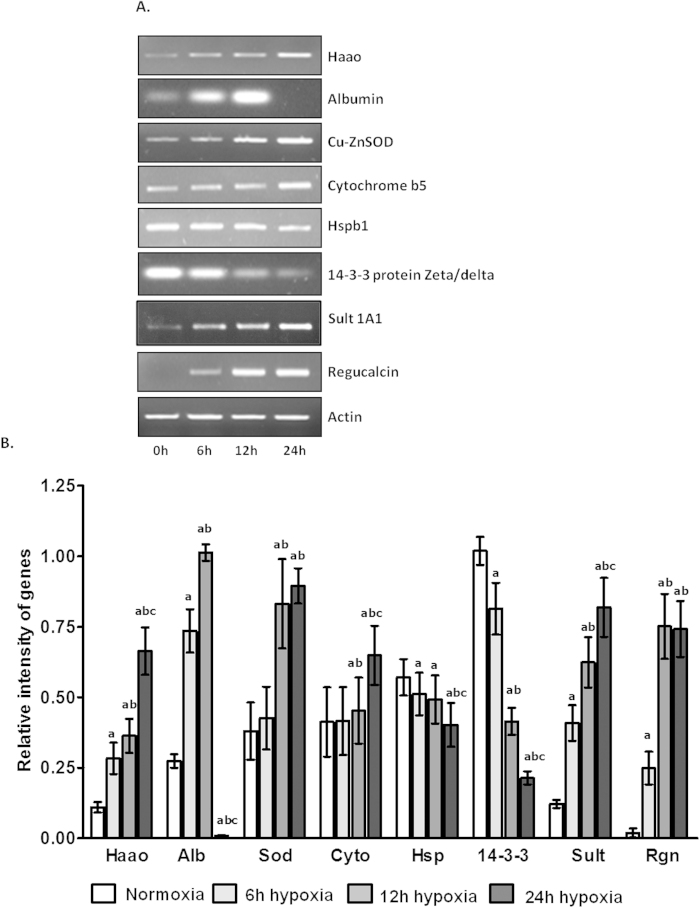
Identification of the expression patterns at different time points after hypobaric hypoxia in lungs at mRNA levels by semiquantitative RT-PCR analysis. Total RNA of rat lungs was prepared separately to synthesize the first- strand cDNA. PCR’s were performed using TaKaRa Taq enzyme and specific primers. β-actin was used as the internal control. Image analysis was performed using Quantity One Software (Bio-Rad, Gel Doc System). Bar graph showed mRNA levels obtained from quantitative densitometry analysis. Data were represented as Mean ± SD of three individual experiments. Differences were considered to be significant when P ≤ 0.05.

The relative differential mRNA expression of eight genes between control and hypobaric hypoxia exposed rats lung were evaluated where the expression of the housekeeping gene actin was used as a reference for normalization. In this study, we validated the expression changes of Haao, Alb, Sod1, Cyb5a, Rgn, Hsb1, Sult 1A1 and Ywhaz in lungs at mRNA level also (Fig. 7). We found that the expression of Haao increased gradually from 6 to 24 h after acute hypobaric hypoxia exposure. The expression of Alb increased gradually from 6 to 12 h but nearly undetectable at 24 h after post hypoxia exposure. The mRNA levels of Sod1, Cyb5a and Sult1A1 were up-regulated gradually from 6 to 24 h. Hsb1 and Ywhaz both were down-regulated in lung after acute hypobaric hypoxia exposure. The mRNA level of Rgn increased 13 fold at 6 h and increased significantly (39 fold) at 12 h, remained at high level after 24 h hypobaric hypoxia exposure as compared to control (0 h) where the expression of Rgn was undetectable.

### Sulfotransferase 1A1 as Biomarker of HAPE

Our PCA analysis showed that 24 hrs time point to be unique in nature. We shortlisted five proteins based on expression value (fold change) at 24 hrs namely Haao, Krt10, Rgn, Sult1a1 and Cyb5a and tested their expression in plasma of human HAPE patients by ELISA. We observed that out of five proteins expression, Sult1A1 was elevated in plasma of HAPE subjects when compared with sea level healthy control subjects as well suggesting its value as a biomarker (Supplementary File 10, Figure S9).

## Discussion

This study was undertaken to identify alterations in the lung proteome caused due to exposure to hypobaric hypoxia. Using an unbiased approach based on proteomic analysis, we have identified a number of proteins that were significantly altered during the hypobaric hypoxia exposure. The term “unbiased” in this study refers to the lack of a priori selection for study of specific known proteins and does not exclude a degree of bias because of the proteomic platform used. Further confidence in the data comes from the detection of proteins that have been reported by others to change, confirmation by western blotting, immunohistochemistry, semiqunatitative RT-PCR and biological plausibility.

We also examined temporal changes in the lung proteome exposed to acute hypobaric hypoxia by generating protein networks that mapped detailed protein interactions. Our approach has several key advantages. By incorporating proteins and biological molecules that were not identified from our proteomics experiments, but are known to be highly connected with other members of the interactome in the lungs, the network analysis correctly predicted a number of critical mediators in the pathogenesis of hypobaric hypoxia induced inflammation/injury. These proteins included HSP 90, JNK, AKT and P38 MAPK. Previous studies showed that NFkB and P38 MAPK regulate key physiologic responses, including neutrophil recruitment and clearance of edema fluid, during the development of lung injury^32^,^33^,^34^. The proteins that were added into the network were predominantly cytokines, intracellular signaling proteins, and transcription factors that exist in extremely low concentrations in the lung. These proteins were not identified in the proteomic analysis because they could be below the threshold of detection for the 2-DE method. The unprompted addition of these proteins with previously identified roles in hypoxia to the in silico network has two important implications. First, it supports the interpretation that the protein networks generated in this study accurately model the proteome of the hypoxia inflamed/injured lung. Second, it suggests that the nodes in the network that have not been well described may also represent novel mediators of hypoxia induced inflammation/injury. The 40 differentially expressed proteins initially identified represent potential biomarkers or indicators of cellular/tissue hypoxia belonging to five major groups which are discussed below.

### Energy Metabolism Related Proteins

The proteins ATP synthase subunit beta (spot 23) and cytochrome b5a (spot 69) that were identified in the study are associated with energy and metabolism. Their altered expression levels are consistent with the altered energy metabolism seen under hypoxic conditions. We have found increased levels of ATP synthase (complex V) subunit after 24 h of hypobaric hypoxia exposure. In mitochondria, this proton pump generates ATP from ADP and organic phosphate using the proton motive force, lowering the membrane potential. Therefore, it seems this increase in ATP synthase may constitute another adaptation towards the maintenance of a lower charge gradient across the mitochondria membrane, in an effort to prevent ROS formation due to hypoxia-normoxia cycles and to high rates of fatty acid β-oxidation^35^. A further interesting protein identified in hypoxic lung was cytochrome b5a which is a highly conserved protein involved in electron transfer between NADH/NADPH and cytochrome P450, participating in the oxidation of a wide array of endogenous and xenobiotic substances^36^. In addition, cytochrome b5 and cytokeratin 17 have been implicated as biomarkers in bronchoalveolar fluid signifying onset of acute lung injury^37^. In the present study, the expression of cytochrome b5a was up regulated (3.5 fold times) after 24 h of hypobaric hypoxia exposure indicating it to be an important oxygen sensing molecule in hypoxic lung. These proteins are involved in aerobic respiration, which is the most efficient metabolic energy pathway. In hypoxia, the up regulation of these proteins likely reflects severe energy deficits in the lung tissue, and these proteins would be quickly activated to produce energy under early hypoxia exposure.

### Signal Transduction Related Proteins

Heat shock proteins are constitutively expressed at low levels in all cells under physiological conditions. Their expression is rapidly induced by various stress factors, including heat, hypoxia, cytotoxic drugs, and radiation^38^. It has been well established that Hsp-b1/Hsp 27 (spots 47 and 52) participates in smooth muscle cell contraction^39^,^40^,^41^. In the present study Hsp-b1 was down regulated after 24 hr of acute hypobaric hypoxia exposure preventing the pulmonary endothelial barrier disruption and decreases the degree of damage in the lungs. We also found a decreased expression of 14-3-3 tau protein which has not been previously reported in hypoxic lung. 14-3-3 proteins can interact with a vast array of different proteins and play a role in diverse functions like cell cycle regulation, signaling transduction and apoptosis^42^. The pathological role of the lower expression of 14-3-3 tau protein under hypoxia remained unclear but we speculate that reduction in 14-3-3 isoforms in acute inflamed lungs may be linked to lung remodeling development such as smooth muscle cell and goblet cell hypertrophy and subepithelial fibrosis. Another protein whose expression was down regulated was annexin A5 (Anxa5, spot 24 and 35) in lung after acute hypobaric hypoxia exposure. Interestingly, the use of Anxa5 as a clinical tool for visualization of cell death has been suggested to be important in monitoring pathologies such as atherosclerosis, myocardial infarction, and cancer^43^.

### Oxidative Stress Proteins

A further interesting protein identified first time in hypoxic lung was regucalcin (spot 33) also known as Senescence marker protein 30 (SMP30). Regucalcin is a multifunctional protein providing protection to cellular functions from age-related deterioration^44^, acts as Ca^2+^ regulator^45^ and anti-oxidants^46^. It also plays a profound role in rescuing cells from cellular injuries such as apoptosis and hypoxia^47^. It was also found that oxidative stress was increased in the brains of SMP30 KO mice without influencing antioxidant enzyme status^48^. SMP30 KO mice also showed senile lung like pulmonary emphysema, and SMP30 protected the lung from oxidative stress associated with aging and smoking^49^. Sheng-qing Li and colleagues reported that the disappearance of SMP-30 at 48 h after acute pulmonary embolism might aggravate the injury of lung tissues^50^. In this study, the expression level of regucalcin was significantly increased (8.7 folds) in lung after 24 h of exposure, suggesting the protective role in response to hypobaric hypoxia and could be of physiological relevance. A novel protein identified first time in hypoxic lung was sulfotransfersae 1A1 (SULT1A1, spot 61). It belongs to a family of phase II detoxification enzymes that catalyze the transfer of the sulfonyl group from 3′ phosphoadenosine 5′ phosphosulfate to a variety of xenobiotics and endogenous compunds such as hormones, neurotransmitters, carcinogens, etc^51^. SULT1A1 can also contribute to increased cancer risk^52^, including breast cancer risk^53^. A recent publication from our lab showed the upregulation of SULT1A1 in plasma of rat exposed to acute hypobaric hypoxia^54^. In present study, it was found that the expression of SULT1A1 in lung tissue was 5-fold increased after 24 hr of acute hypobaric hypoxia. On the basis of their specific roles and diagnostic prospects, expression pattern of SULT1A1 was verified in plasma samples of control sea levels and HAPE patients. Comparison of plasma samples from 10 HAPE patients and 10 healthy sea level controls by ELISA analysis confirmed that concentration of SULT1A1 was significantly increased in the plasma of HAPE patients as compared to the sea level controls (Supplementary File 10, Figure S9). The mean plasma SULT1A1 concentration was 4870 ± 2155 ng/mL (Mean ± SD) in HAPE patients versus 169.3 ± 91.13 ng/mL in sea level controls (p < 0.0001). Our data thus indicates that the pattern of SULT1A1 protein upregulation in HAPE patients was on similar lines as we observed in rat model.

### Structural Proteins involved in Cytoskeleton Organization

The hypobaric hypoxia exposure response is usually accompanied by reorganization of the cytoskeleton. During our experiment, changes in the abundance of several cytoskeletal proteins were noted (1 and 2-actin cytoplasmic, keratin type I cytoskeletal 19, myosin light chain-4 and keratin type I cytoskeletal 10). The actin cytoskeleton (spot 15–21 and 30) is important for many cellular functions, including cell motility, structure and integrity. Both 1 and 2- actins show a parallel expression pattern in our experimental model that is, at 24 h, the levels significantly fell after post-hypobaric hypoxia exposure as compared to control. In this sense, other authors have reported that lowering O2, and probably in part through HIF-1, alters the expression of actins^55^. Our results may suggest the notion that the cytoskeleton of lung cells is highly sensitive to hypobaric hypoxia and indicates that this situation may induce a cytoskeleton restructuring, possibly to maintain cellular functionality. Keratin, type I cytoskeletal 19 (KRT19, spot 22) is strongly expressed by normal simple bronchial and respiratory epithelium. Although CK19 is a part of the cytoskeleton, a soluble fragment of this polypeptide can be released and assayed in the blood as CYFRA 21–1. The release of it may result in the downregulation of KRT 19 and cellular apoptosis^56^. So the downregulation of CK19 might indicate prevailing activities of apoptosis and injury in lung tissues after hypobaric hypoxia exposure. During the course of our work, we also observed an increased (8.7 fold) in keratin, type I cytoskeletal 10 (KRT10, spot 31) in hypoxic lung at 24 h indicating damage to the cytoskeleton, which consequently affects the cell morphology and function. Myosin light chain 4 (MYL4, spot 51) is a component of smooth muscle thick filaments. Thongboonkerd *et al.* reported that smooth muscle myosin was downregulated in sustained hypoxia^57^. In present study myosin light chain 4 expression was downregulated, which is in accordance with the findings of Thongboonkerd *et al.*

### Ribosomal Proteins

40S ribosomal protein SA (RPSA, spot 26 and 27) is also known as 37 kDa laminin receptor precursor and p40 protein. It is a bifunctional protein, being both an essential component of ribosomes and a receptor for laminin and other ligands^58^. In our experimental model, RPSA was downregulated at the protein level after hypobaric hypoxia treatment. The inhibition of protein synthesis and the conservation of energy is advantageous for hypoxic lungs because a decreased translational rate will consequently reduce oxygen consumption. Reduced oxygen consumption by translational arrest has been reported to be logical and established mechanism for the reduction of cellular injury, but not after hypoxia^59^. Consistent with the reduction in protein synthesis, our data suggest that the downregulation of RPSA protein may contribute to hypoxia-mediated global translation attenuation in lungs.

The probable tRNA pseudouridine synthase 1 is thought to play a role in the initiation of translation^60^. Recently, it was discovered that this protein can be induced by stress, thus proposing a regulatory role in survival, and that, by mediating a nonsense-to-sense codon conversion, it may represent new means of generating coding or protein diversity^61^. This protein (TRUB1, spot 32, 40) has not been identified previously in hypoxic lung.

### Antioxidant and Acute phase Proteins

Hemopexin (HPX, spot 2, 3) is a positive acute phase protein that binds heme and transports it to liver for breakdown recovery and prevents heme-mediated oxidative damage and heme-bound iron- loss^62^. More recently, a publication from our lab reported the upregulation of HPX in plasma of high altitude natives suggesting its important physiological role as an antioxidant^63^. In our study, HPX was upregulated which is in accordance with the previous findings.

Serum albumin (ALB, spot 5–8, 10, 11) known as negative acute phase protein is one of the major antioxidants in the respiratory tract lining fluid, which also includes mucin, superoxide dismutase, glutathione, uric acid and ascorbic acid. The free thiol group at ^34^cysteine makes albumin the greatest quantitative scavenger of reactive species in serum^64^. As has been suggested in chronic obstructive pulmonary disease, the capacity for oxidative modification of albumin serves to buffer oxidative stress^65^. In the present work, we focused on the antioxidant activity of albumin because oxidative stress is thought to play a significant role in the pathogenesis of high altitude related disease^66^. Decreased levels of serum albumin after 24 h of hypobaric hypoxia exposure could therefore contribute to the excessive accumulation of oxidants which would lead to enhanced expression of pro-inflammatory mediators, inactivation of anti-proteinases and ultimately oxidative tissue injury.

A further interesting protein identified was copper/zinc superoxide dismutase (SOD1, spot 62, 63) which is located in epithelial cells, fibroblasts and alveolar macrophages^67^. Its main function is to scavenge O_2_^−^, inhibiting redox generation of ROS and RNS^67^. Overexpression of SOD1 in transgenic mice has been shown to be highly protective in multiple models of brain injury including trauma, ischemia/repurfusion injury, mitochondrial inhibition and methamphetamine induced neurotoxicity^68^,^69^. In our study, SOD1 was upregulated and this corresponds well with the previous findings, confirming the relevance of this molecule as a defense tool for the lung against hypobaric hypoxia-induced damage.

During the course of our study, we also observed an increased in protein disulfide-isomerase A3 (PDIA3, spot 12, 13) abundance in hypoxic lung after 24 h. This protein has both a disulfide-isomerase activity which helps the correct formation of disulfide bridges between cysteine residues and chaperone activity, preventing proteins from misfolding in endoplasmic reticulum^70^. In present study, PDIA3 was upregulated suggesting protective role in the initial response to the ER stress resulting from hypobaric hypoxia exposure.

### Study Limitations

We have noted some important limitations that need to be overcome to increase the sensitivity and enhance information capture. The identification of low-abundance proteins may often be hindered by the highly abundant ones, which mask them. Important biomarker information could thus be lost because there is no PCR equivalent available to amplify proteins. Since most of the proteins have multiple isoforms that can differ in electrophoretic mobility in 2D gels affecting the accuracy and reproducibility of single protein identification. Moreover, the smaller dynamic range of the method also results in identification of limited number of proteins. Secondly, although our work has provided some important clues, further studies are still needed to elucidate the detailed roles of these differentially expressed proteins during acute hypobaric hypoxia exposure.

## Conclusions

Previous studies have reported specific changes in physiological parameters including fluid leakage, memory/cognition that can be distinctively observed by 18–24 hrs of hypobaric hypoxia exposure. Since the final outcome of the high altitude exposure majorly depends on the duration of stay, it is important to decipher the temporal proteomic changes in lung tissue for better understanding of sequential cascade of molecular events regulated by hypobaric hypoxia. Moreover, comparison of proteome profiles will provide a molecular insight into differential hypoxia response of lung proteins in rats. The present study thus reveals, for the first time, major alterations in expression level of newly identified proteins, regucalcin, sulfotransferase 1A1, Keratin, type I cytoskeletal 10 and other proteins. The identified proteins in hypoxic lung are involved in specific biological processes (inflammation, immunity, oxidative stress, haemostasis and signalling) and potentially implicated in the pathogenesis of hypoxia induced lung injury/inflammation. To best of our knowledge, for the first time we report SULT1A1 correlation with HAPE, which could be considered for use as a novel biomarker to diagnose HAPE. The confirmation of this specific protein in the plasma of HAPE patients and healthy control subjects has demonstrated their discrimatory power of HAPE detection. Although further validation in a larger sample set is necessary for biomarker discovery. Additionally, such discoveries promote new directions of thought and research regarding the pathophysiology of high altitude hypoxia related lung disease like HAPE, pathogenesis of complications involved in it, potential treatments targeting these proteomically identified aberrations, and earlier, and more specific, screening and diagnostic modalities. Future investigation in this area over the next five years holds promise for devising new biomarkers to predict prognosis of lung disease, stratify risk and provide surrogate outcome markers for future clinical trials in lung disease related to hypobaric hypoxia. Such biomarkers could help to guide risky curative therapies and also to accelerate drug development to reduce the burden of human suffering from high altitude illness.

## Additional Information

**How to cite this article**: Ahmad, Y. *et al.* The proteome of Hypobaric Induced Hypoxic Lung: Insights from Temporal Proteomic Profiling for Biomarker Discovery. *Sci. Rep.* **5**, 10681; doi: 10.1038/srep10681 (2015).

## Supplementary Material

Supplementary Information

Supplementary Dataset 1

Supplementary Dataset 2

Supplementary Dataset 3

Supplementary Dataset 5

Supplementary Dataset 6

Supplementary Dataset 7

Supplementary Dataset 8

Supplementary Dataset 9

Supplementary Dataset 10

Supplementary Dataset 11

Supplementary Dataset 12

Supplementary Dataset 13

Supplementary Dataset 15

Supplementary Dataset 16

Supplementary Dataset 17

Supplementary Dataset 18

Supplementary Dataset 19

Supplementary Dataset 20

Supplementary Dataset 21

Supplementary Dataset 22

Supplementary Dataset 26

Supplementary Dataset 27

Supplementary Dataset 29

Supplementary Dataset 30

Supplementary Dataset 31

Supplementary Dataset 32

Supplementary Dataset 33

Supplementary Dataset 34

Supplementary Dataset 35

Supplementary Dataset 36

Supplementary Dataset 37

Supplementary Dataset 38

Supplementary Dataset 39

Supplementary Dataset 40

Supplementary Dataset 44

Supplementary Dataset 47

Supplementary Dataset 51

Supplementary Dataset 52

Supplementary Dataset 61

Supplementary Dataset 63

Supplementary Dataset 64

Supplementary Dataset 69

## Figures and Tables

**Table 1:** Primer Sequences and the Size of Products of nine genes.

**Gene Name**	**GenBank Accession No.**	**Primer Sequence (Forward and Reverse)**	**Amplicon size**	**Annealing Temp**
3-hydroxyanthranilate 3,4-dioxygenase (Haao)	NM_020076	F- 5’- GGAGGCCCCAATACCAGGA -3’	120 bp	54 °C
		R- 5’- TATAGGCACGTCCCGGTGTT-3’		
Rattus norvegicus albumin (Alb)	NM_134326	F- 5’- CTTCAAAGCCTGGGCAGTAG-3’	188 bp	56 °C
		R- 5’- TGGAGATAGTGGCCTGGTTC -3’		
Superoxide dismutase 1, soluble (Sod1)	NM_017050	F- 5’- CCACTGCAGGACCTCATTTT -3’	216 bp	56 °C
		R- 5’- CACCTTTGCCCAAGTCATCT -3’		
Cytochrome b5 reductase 3 (Cyb5r3)	NM_138877	F- 5’- CTGTGTCTAGTGATGATGAC-3’	142 bp	58 °C
		R- 5’- CGGAATTCAATGGTGTCTCC-3’		
Regucalcin (senescence marker protein-30) (Rgn)	NM_031546	F- 5’- TCAAAGACTGTCTGCCGATG -3’	92bp	56 °C
		R- 5’- GACTGTCGAAGTGCCACTGA -3’		
Heat shock protein 1 (Hspb1)	NM_031970	F- 5’-TGTCAGAGATCCGACAGACG-3’	220 bp	60 °C
		R- 5’-GACAGGGAAGAGGACACCAA-3’		
Tyrosine 3- monooxygenase/tryptophan 5-monooxygenase activation protein, zeta (14-3-3 protein Zeta/delta)	NM_013011	F- 5’- TTGAGCAGAAGACGGAAGGT -3’	201 bp	56 °C
		R- 5’- GAAGCATTGGGGATCAAGAA-3’		
Sulfotransferase 1A1 (Sult 1A1)	NM_031834	F-5’- CATGGAGTTCTCCCGTCCAC-3’	344 bp	58 °C
		R- 5’- GCAGACTCTGAGGGAGCAAG-3’		
Actin	NM_019212	F- 5′-TGCGCGACATCAAAGAGAAG-3′	250bp	58 °C
		R- 5′-GTTGTTGGCGTACAGGTCCT-3′		

**Table 2:** List of lung proteins altered in response to hypobaric hypoxia.

**Spot ID**	**UniProt Accession No.**	**Protein Description**	**Mascot Score**	**Calculated MASS/pI**	**Observed MASS/pI**	**% Cov.**	**Peptide Match**	**Gene Name**	**Protein Identification**	**Quantitative Fold Change**		
										**6 H**	**12 H**	**24 H**
2	P20059	Hemopexin	77	52.06/7.6	88/5.85	28%	10	**Hpx**	PMF,PFF		▲1.3^c^	▲1.9^a^
3	P20059	Hemopexin	79	52.06/7.6	84/5.97	28%	10	**Hpx**	PMF,PFF	▲1.2^c^	▲1.4^b^	▲1.8^a^
5	P02770	Serum albumin	135	70.68/6.09	83/6.26	30%	16	**Alb**	PMF,PFF	▲1.5^b^	▲1.3^b^	
6	P02770	Serum albumin	133	70.68/6.09	85/6.17	27%	13	**Alb**	PMF,PFF	▲1.6^a^	▲1.5^b^	▲1.2^c^
7	P02770	Serum albumin	260	70.68/6.09	84/6.07	45%	22	**Alb**	PMF.PFF	▲1.5^b^	▲1.4^b^	
8	P02770	Serum albumin	101	70.68/6.09	82/5.96	24%	14	**Alb**	PMF.PFF	▲1.5^b^	▲1.4^b^	▼0.8^c^
9	Q499M5	Ankyrin repeat domain-containing protein 16	52	41.33/6.16	76/5.94	24%	6	**Ankrd16**	PMF	▲1.3^c^	▲2.9^a^	▲3.7^a^
10	P02770	Serum albumin	68	70.68/6.09	82/5.89	21%	9	**Alb**	PMF.PFF	▲1.8^a^	▲1.5^b^	▼0.6^b^
11	P02770	Serum albumin	33	70.68/6.09	83/5.81	14%	6	**Alb**	PMF.PFF	▲2.1^a^	▲1.4^b^	▼0.6^b^
12	P11598	Protein disulfide-isomerase A3	57	57.04/5.88	64/6.13	21%	9	**Pdia3**	PMF.PFF			▲1.7^a^
13	P11598	Protein disulfide-isomerase A3	100	57.04/5.88	65/5.88	3%	1	**Pdia3**	PFF	▲1.3^c^	▲1.2^c^	▲1.5^a^
15	P60711	Actin, cytoplasmic 1	130	42.05/5.29	44/5.39	46%	10	**Actb**	PMF,PPF		▲1.2^c^	▼0.5^a^
16	P60711	Actin, cytoplasmic 2	89	42.11/5.3	45/5.31	40%	11	**Actg1**	PMF	▲1.2^c^	▲1.3^c^	
17	P60711	Actin, cytoplasmic 2	135	42.11/5.3	45/5.26	63%	15	**Actg1**	PMF,PPF	▲1.4^b^	▲1.5^a^	
18	P60711	Actin, cytoplasmic 1	64	42.05/5.29	45/5.2	2%	1	**Actb**	PPF	▲1.4^b^	▲1.5^a^	▼0.5^a^
19	P60711	Actin, cytoplasmic 1	106	42.05/5.29	44/5.15	42%	10	**Actb**	PMF	▲1.4^b^	▲1.4^a^	▼0.6^b^
20	P60711	Actin, cytoplasmic 1	77	42.05/5.29	43/5.12	25%	7	**Actb**	PMF		▲1.8^a^	▲1.6^a^
21	P60711	Actin, cytoplasmic 1	62	42.05/5.29	43/5.15	36%	7	**Actb**	PMF		▲2.0^a^	▲1.5^b^
22	Q63279	Keratin, type I cytoskeletal 19	130	44.61/5.21	43/5.21	33%	15	**Krt19**	PMF,PPF		▲1.4^b^	▼0.4^a^
23	P10719	ATP synthase subunit beta	40	56.32/5.19	56/4.94	17%	6	**Atp5b**	PMF,PPF	▼0.7^b^	▲1.4^b^	▲2.5^a^
26	P38983	40S ribosomal protein SA	67	32.92/4.80	44/4.67	36%	7	**Rpsa**	PMF,PPF		▼0.8^c^	▲1.2^c^
27	P38983	40S ribosomal protein SA	67	32.92/4.80	44/4.75	36%	7	**Rpsa**	PMF,PPF			▲1.5^a^
30	P60711	Actin, cytoplasmic 1	43	42.05/5.29	39/5.55	20%	5	**Actb**	PMF,PPF	▲1.2^c^	▲1.2^c^	▼0.5^**a**^
31	Q6IFW6	Keratin, type I cytoskeletal 10	48	56.70/5.10	42/5.55	1%	1	**Krt10**	PPF			▲8.7^a^
32	Q5M934	Probable tRNA pseudouridine synthase 1	51	36.61/8.86	38/5.17	14%	6	**Trub1**	PMF	▼0.5^b^	▲1.8^a^	▲3.7^a^
33	Q03336	Regucalcin	110	33.94/5.27	37/5.17	43%	11	**Rgn**	PMF,PPF	▲1.4^b^	▲1.5^a^	▲8.7^a^
34	P14668	Annexin A5	115	35.77/4.93	34/4.84	38%	14	**Anxa5**	PMF,PPF			▼0.3^a^
35	P14668	Annexin A5	123	35.77/4.93	34/4.9	38%	11	**Anxa5**	PMF,PPF		▲1.2^c^	▼0.4^a^
37	P63102	14-3-3 protein zeta/delta	55	28.15/4.81	30/4.66	7%	1	**Ywhaz**	PPF			▼0.5^a^
38	P63102	14-3-3 protein zeta/delta	107	27.93/4.73	30/4.72	7%	1	**Ywhaz**	PPF			▼0.6^b^
39	Q64537	Calpain small subunit 1	57	28.67/5.29	29/5.21	28%	6	**Capns1**	PMF,PPF	▼0.7^b^		▼0.8^c^
40	Q5M934	Probable tRNA pseudouridine synthase 1	53	36.61/8.86	33/5.22	23%	7	**Trub1**	PMF	▼0.8^c^	▼0.6^b^	▼0.4^a^
44	P46953	3-hydroxyanthranilate 3,4-dioxygenase	66	32.85/5.57	33/5.54	22%	7	**Haao**	PMF,PPF	▲1.3^a^	▲1.4^b^	▲10.2^a^
47	P42930	Heat shock protein beta-1	97	22.94/6.12	28/5.47	36%	8	**Hspb1**	PMF		▲1.3^c^	▼0.5^a^
51	P17209	Myosin light chain 4	100	21.38/4.96	25/4.95	7%	1	**Myl4**	PPF	▼0.6^b^	▼0.5^a^	
52	P42930	Heat shock protein beta-1	118	22.94/6.12	28/5.95	41%	9	**Hspb1**	PMF,PPF		▼0.8^c^	▼0.2^a^
61	P17988	Sulfotransferase 1A1	71	34.17/6.37	36/6.69	35%	9	**Sult1a1**	PMF,PPF	▲1.2^c^		▲5.2^a^
62	P07632	Superoxide dismutase [Cu-Zn]	81	16.07/5.88	19/6.14	38%	6	**Sod1**	PMF,PPF	▲1.3^b^		▲2.1^a^
63	P07632	Superoxide dismutase [Cu-Zn]	81	16.07/5.88	15/6.21	38%	6	**Sod1**	PMF,PPF	▲1.2^c^		
69	P00173	Cytochrome b5	52	15.35/4.90	14/5.25	41%	4	**Cyb5a**	PMF,PPF	▲1.4^b^		▲3.5^a^

The identified proteins were indicated along with Swiss-Prot accession number and expression level in terms of percentage of difference of an individual spot between hypobaric hypoxia exposed at each exposure time point (6 h, 12 h and 24 h) in comparisons to normoxic controls. Triangles pointed upward and downward indicate an increase or decrease respectively, of a spot intensity in exposed as compared with their normoxic control littermates. (‘a’ p < 0.001, ‘b’ p < 0.01, and ‘c’ p < 0.05).

**Table 3:** Altered pathways in response to hypoxia (Ingenuity Pathway Analysis, p ≤ 0.05).

**6h**	**12h**	**24h**
GAP Junction Signalling	GAP Junction Signalling	Branched–chain α-keto acid Dehydrogenase Complex
NRF-2 Mediated Oxidative stress Response	VEGF Signalling	Superoxide- Radicals Degradation
VEGF Signalling	Acute Phase Response Signalling	14-3-3 mediated Signalling
Superoxide- Radicals Degradation	NRF-2 Mediated Oxidative stress Response	Mitochondrial Dysfunction
Mitochondrial Dysfunction	ILK Signalling	Acute Phase Response Signalling
Acute Phase Response Signalling	Actin Cytoskeleton Signalling	NRF-2 Mediated Oxidative stress Response
Actin Cytoskeleton Signalling	Antigen Presentation Pathway	Cell Cycle G2/M DNA Damage Checkpoint Regulation
Apoptosis Signalling	Antioxidant Action of Vitamin C	Apoptosis Signalling
Antioxidant Action of Vitamin C	14-3-3 mediated Signalling	VEGF Signalling
14-3-3 mediated Signalling	Mitochondrial Dysfunction	p38 MAPK Signalling
IL-12 Signalling and Production in Macrophages	Production of Nitric Oxide and reactive Oxygen Species in Macrophages	PI3K/AKT Signalling
Calcium Signalling	Thrombin Signalling	Protein Ubiquitination Pathway
