# Author Correction: The proteome of Hypobaric Induced Hypoxic Lung: Insights from Temporal Proteomic Profiling for Biomarker Discovery

**DOI:** 10.1038/s41598-022-06169-9

**Published:** 2022-02-01

**Authors:** Yasmin Ahmad, Narendra K. Sharma, Mohammad Faiz Ahmad, Manish Sharma, Iti Garg, Mousami Srivastava, Kalpana Bhargava

**Affiliations:** 1 Defence Institute of Physiology & Allied Sciences (DIPAS), DRDO, Ministry of Defence, Timarpur, Delhi, India; 2 School of Biotechnology, JNU, New Delhi, India

Correction to: *Scientific Reports* 10.1038/srep10681, published online 29 May 2015

This Article contains an error in Figure 5B. As a result of an error in figure assembly, the image provided for Cyb5a staining at 12h of hypoxia is incorrect. The correct Figure 5 and accompanying legend appear below.

**Figure 5:**
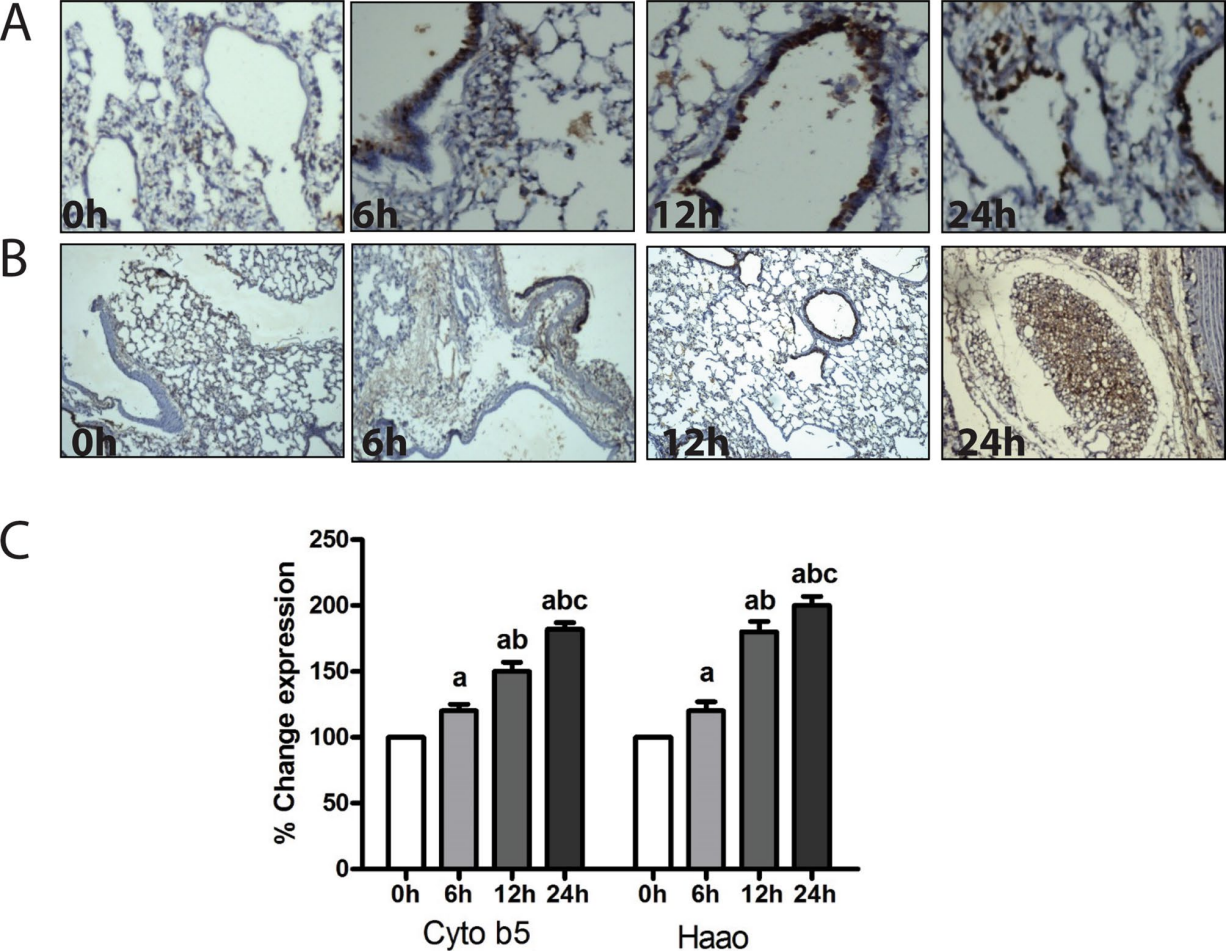
Immunohistochemical analysis of Haao and Cyb5a in rat lungs. (**A**) Immunostaining with an antibody to Haao of normoxic lung (0 h) compared with lung from hypoxic rats treated at different time points (6 h, 12 h and 24 h). Similarly, (**B**) immunostaining with an antibody to Cyb5a of normoxic lung (0 h) compared with hypoxic lung of rats treated at different time points (6 h, 12 h and 24 h). The results showed no positive immunoreactivity for Haao and Cyb5a in control rats (0 h) but Haao and Cyb5a stained strongly in hypoxic lung sections (6 h, 12 h and 24 h). Figure 5 (**C**) showed significantly higher levels of Haao and Cyb5a in hypoxia exposed lung tissues. Data were represented as Mean ± SD of three individual experiments. ‘a’ refers as P ≤ 0.05 with compare to normoxia, ‘b’ refers as P ≤ 0.05 with compare to 6 h hypoxia and ‘c’ refers as P ≤ 0.05 with compare to 12 h hypoxia.

This change does not affect the conclusions of the Article.

