# Supplementary material for: The proteome of Hypobaric Induced Hypoxic Lung: Insights from Temporal Proteomic Profiling for Biomarker Discovery
Source: Sci Rep. 2015 May 29;5:10681. doi: 10.1038/srep10681 (PMC4448130; doi:10.1038/srep10681)
Supplement: Supplementary Information [file srep10681-s1.doc]

**The proteome of Hypobaric Induced Hypoxic Lung: Insights from Temporal Proteomic Profiling for Biomarker Discovery**

Yasmin Ahmad, Narendra K. Sharma, Mohammad Faiz Ahmad, Manish Sharma, Iti Garg, Mousami Srivastava and Kalpana Bhargava

**Supplementary File 1, Table S1: Physical Characteristics of the Subjects**

|  | Control Subjects (n=10) | Patients with HAPE (n=10) |  |  |
| --- | --- | --- | --- | --- |
| Age (yr) | 28.3±1.4 | 27.6±1.4 |  |  |
| Sex | Male | Male |  |  |
| Height | 169.7±1.3 | 170.6±1.3 |  |  |
| Weight | 63.4±1.4 | 60.1±1.3 |  |  |
| BMI | 22.03±0.7 | 20.66±0.5 |  |  |
|  |  |  |  |  |
| **Value: Mean±SD** | |  |  |  |

**Supplementary File 2, Figure S2**

**Normoxia, 0 h**

**
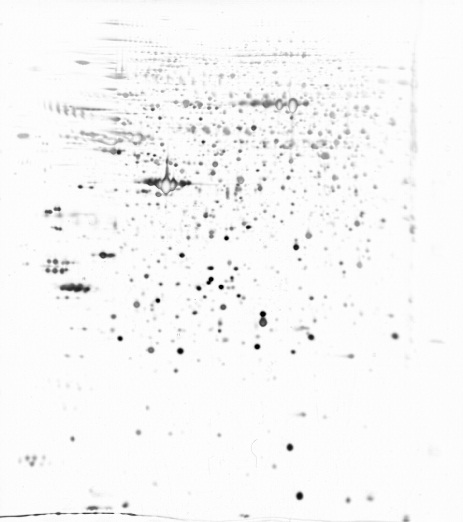
**

**Supplementary File 3, Figure S3**

**Hypoxia, 6 h**

**
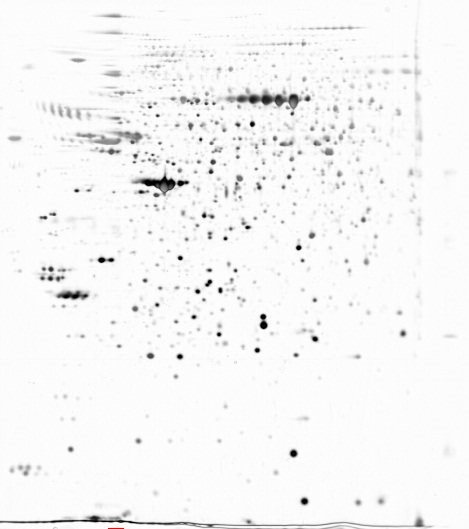
**

**Supplementary File 4, Figure S4**

**Hypoxia, 12 h**

**
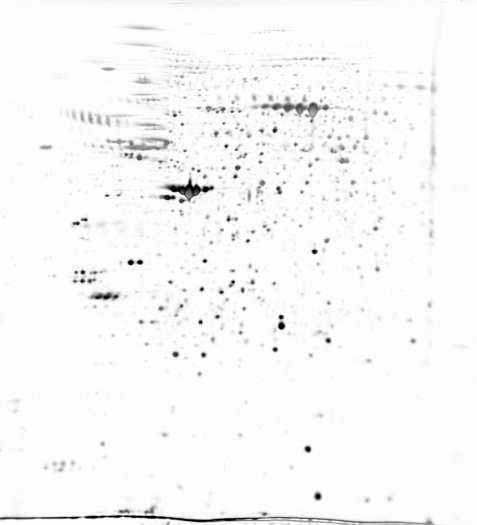
**

**Supplementary File 5, Figure S5**

**Hypoxia, 24 h**

**
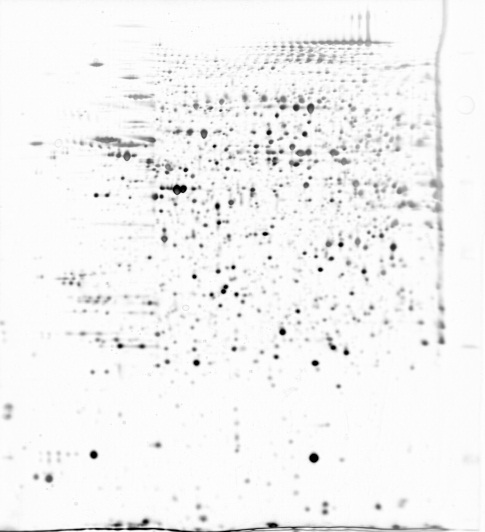
**

**Supplementary File 6, Table S2**

**Signaling Pathway Networks That Invole Hypoxia Lung Mapping Proteins**

| **Networks**  **(Time Duration)** | **Nodes (Genes ; Proteins) in Network** | **Score** | **Identified Nodes (Proteins)** | **Top Functions** |
| --- | --- | --- | --- | --- |
| 6 h  12 h  24 h | Acetic acid, ACTB, ACTG1, Actin, Akt, LB, APP, asparagine, ATP5B, ATP6V1G2, APNS1, CYB5A, DSG3, EPS8L1, EPS8L2, GAS8, hemoglobin, HN1, HPX, Hsp90, Insulin, L-leucine, MYLK3, NCALD, NFkB (complex), PDGF (family), PDIA3, HACTR1, PLS1, PROSER2, SH3BGRL3, SOD1, TMOD2, Tpm1, TRUB1  ACTB, ACTG1, Actin, ALB, ANXA5, ATP5B, Brdt, Cald1, COTL1, DCTN6, Dmd, EPS8L1, EPS8L2, ERK1/2, F Actin, GAS8, HIP1R, HPX, HSPB1, Insulin, Jnk, KRT19, L-leucine, NCALD, NFkB (complex), P38 MAPK, PDGF (family), PDIA3, PHACTR1, PLS1, RPSA, RUSC1, SPTBN2, SSH1, XPO6  ACTB, ACTIN, Akt, ALB, ANXA5, ATP5, ATP5B, ATP6V1G2, CAPNS1, caspase, CYB5A, DSG3, ERK1/2, GUK1, hemoglobin, Histone h4, HN1, HPX, Hsp90, HSP, HSPB1, IgG, IL36RN, Insulin, Jnk, KRT10, KRT19, NFkb (Complex)), P38 MAPK, PDIA3, RPSA, SOD1, Tpm1, Ubiquitin, YWHAZ | 29  26  41 | 11  10  15 | Cellular Assembly and Organization, Hematological Disease, Immunological Disease  Hematological Disease, Immunological Disease, Inflammatory Disease  Cellular Components,  Hematological Disease, Immunological Disease |

**Supplementary File 7, Figure S6**

**
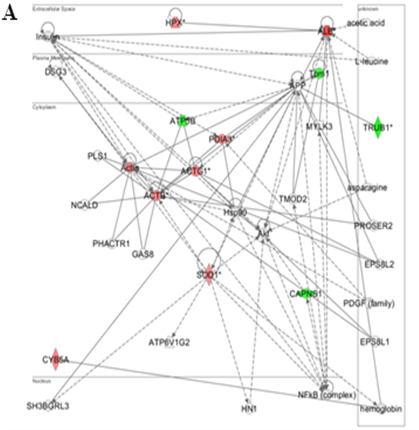

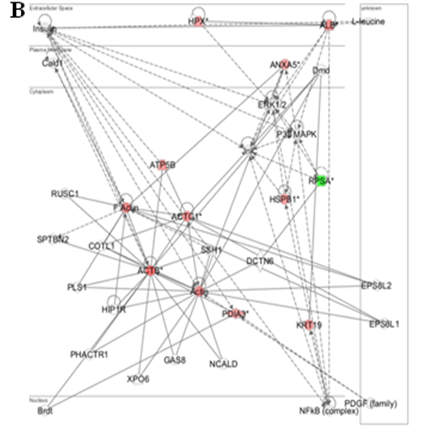
**

**
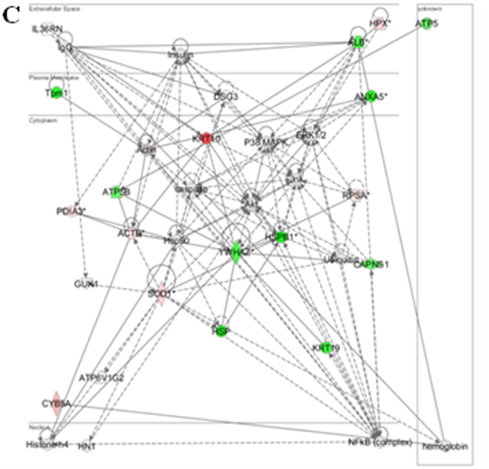
**

Schematic representations of the signalling network of differentially expressed proteins at different exposure time (A) 6 h (B) 12 h and (C) 24 h. The figures was created using Ingenuity and shows the connectivity between nodes and hubs. Solid lines indicate direct interaction. Dashed lines indicate indirect interaction. Arrows indicate stimulation. Bars indicate inhibition. The proteins are shown in a red to green color scale, corresponding to their relative abundance in the rat lung samples at different exposure time compared with control noromoxic lung used in 2D-E analysis. To enhance the connectivity of the network, proteins and other biological compounds that were not identified in the proteome analysis, but had known interactions with the identified proteins were added using the ingenuity program (colorless).

**Supplementary File 8, Figure S7**

**A.**

**
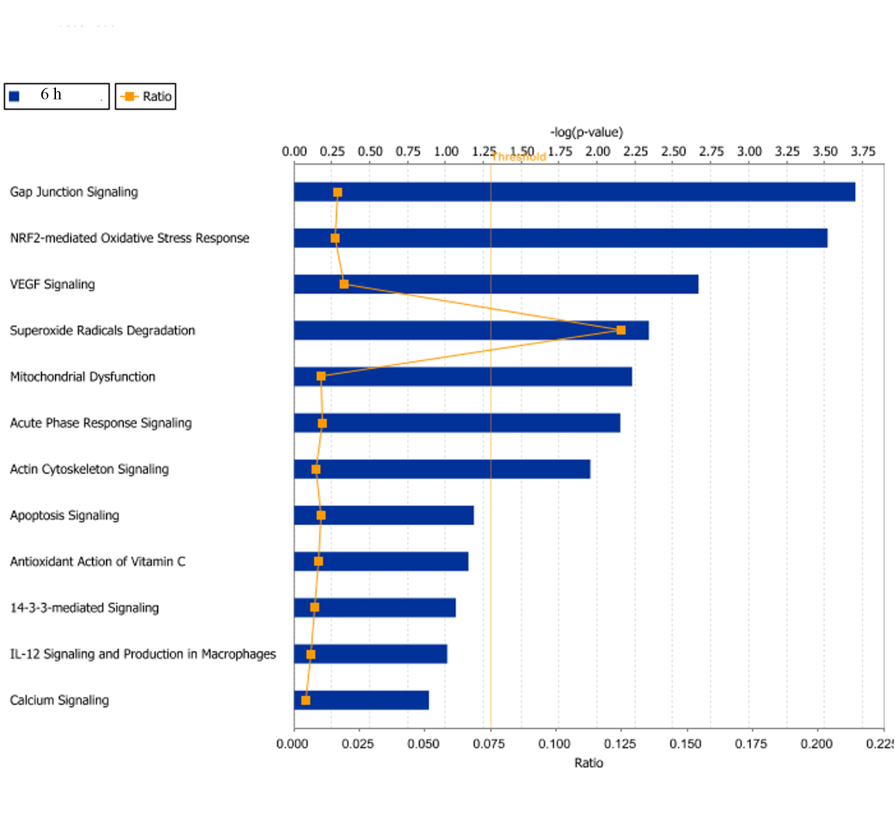
**

**B.
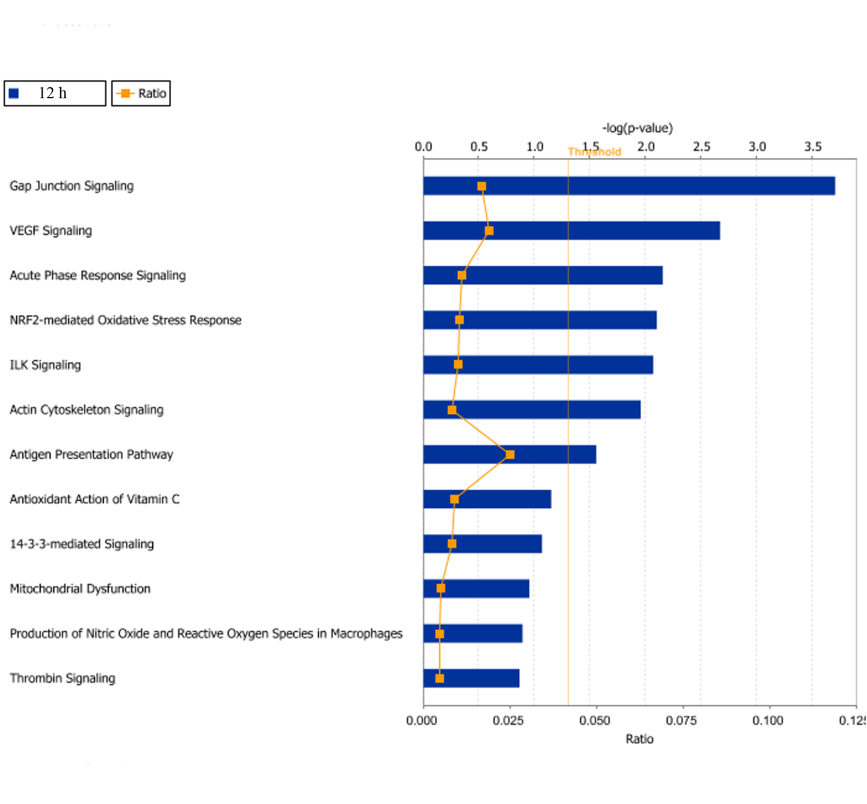
**

**C.**

**
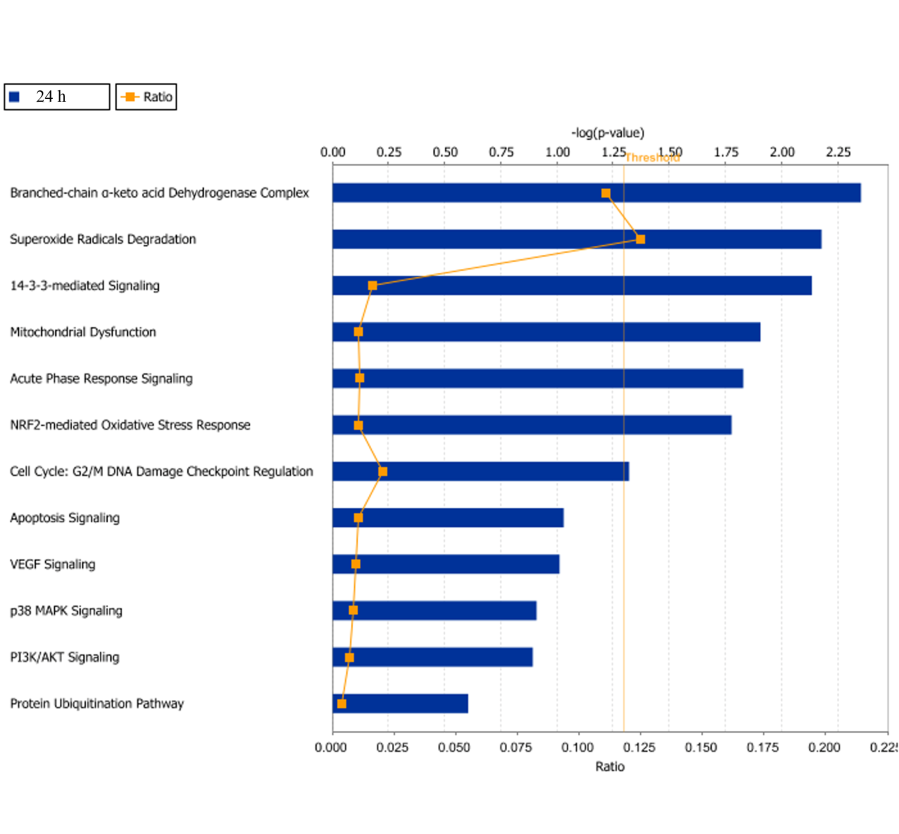
**

Significant canonical pathways (p≤0.05) for proteins differentially expressed in hypoxic lung at different exposure time points (A) 6 h (B) 12 h and (C) 24 h. The negative of the log_10_ (p-value) and ratio (number of focus molecules involved in the pathway/total number of molecules in the pathway) are plotted on the primary and secondary Y-axis respectively. Pathways are indicated by blue columns and the corresponding ration is indicated by a solid line joined by squares.

**Supplementary File 9, Figure S8**

Principal components analysis (PCA) was used to group the lung samples based on their variances in protein expression. This analysis showed that the hypoxic treated and noromoxic treated lung samples cluster into distinct groups in the proteome space. PCA was not able to discriminate control, 6 h exposure and 12 h exposure groupings from one another. In contrast, exposure to hypobaric hypoxia for 24 hours led to dramatic alterations in the protein expression.

**Supplementary File 10, Figure S9**

**Plasma Sulfotransferase 1A1**

**
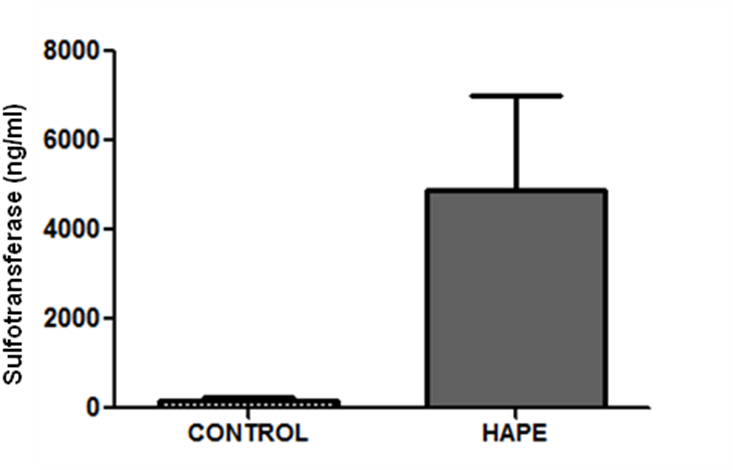
**

Determination of plasma concentration of SULT1A1 in healthy controls and patients with HAPE by ELISA analysis. The mean plasma SULT1A1 concentration was 4870 ± 2155 ng/mL (Mean ± SD) in HAPE patients versus 169.3 ± 91.13 ng/mL in sea level controls (p <0.0001).


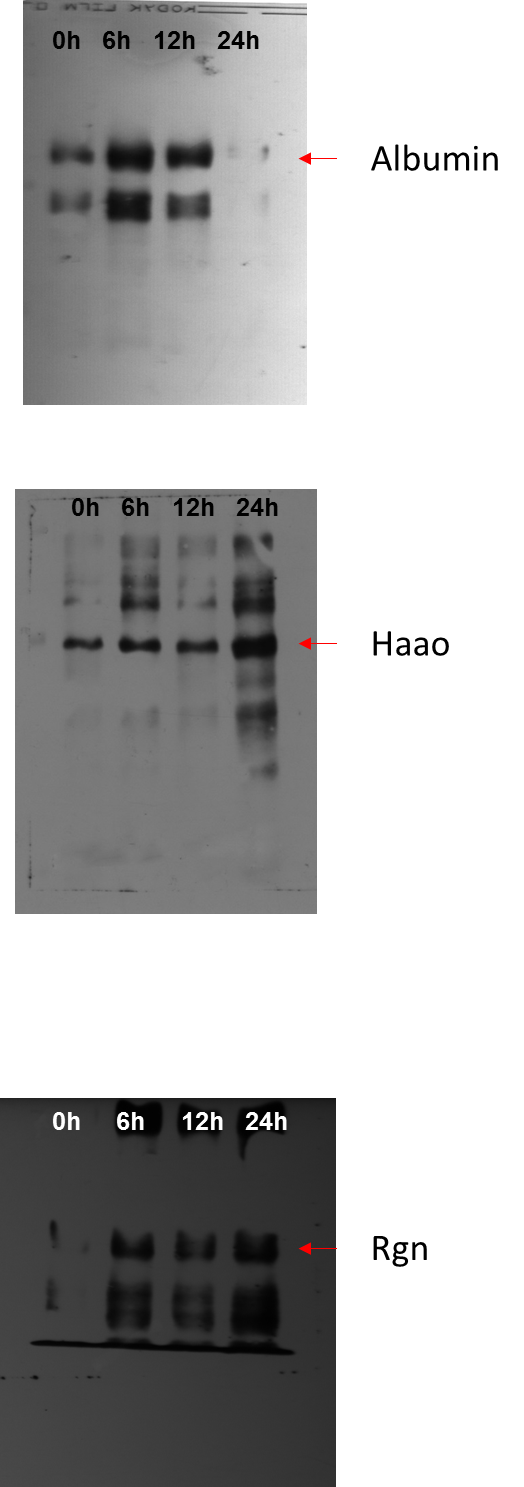


**
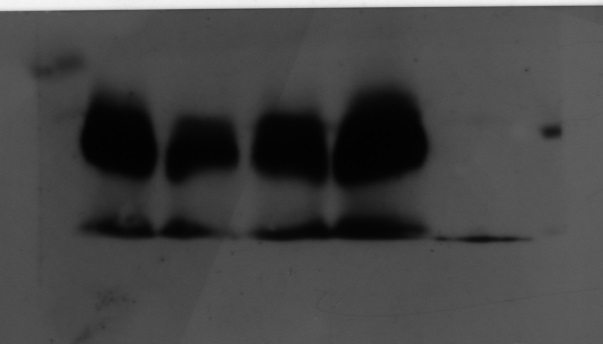
Full Size Uncropped Western**

Cyto b5

**Supplementary File 11**

**Above are the full size uncropped Western Blots**

**Supplementary File 12, Table S3**

**: Hyperlink copy of MASS-SPEC data**

| Spot ID | PMF | PPF |
| --- | --- | --- |
| 2 | [HEMO_RAT](http://www.matrixscience.com/cgi/protein_view.pl?file=../data/20110616/FtteInTeS.dat&hit=1) | [HEMO_RAT](http://www.matrixscience.com/cgi/protein_view.pl?file=../data/20110613/FtteiaSOh.dat&hit=HEMO_RAT&db_idx=1&px=1&ave_thresh=24&_ignoreionsscorebelow=0&report=20&_sigthreshold=0.05&_msresflags=1025&_msresflags2=2&percolate=-1&percolate_rt=0) |
| 3 | [HEMO_RAT](http://www.matrixscience.com/cgi/protein_view.pl?file=../data/20110616/FtteInTeL.dat&hit=1) | [HEMO_RAT](http://www.matrixscience.com/cgi/protein_view.pl?file=../data/20110613/FtteifcaT.dat&hit=HEMO_RAT&db_idx=1&px=1&ave_thresh=24&_ignoreionsscorebelow=0&report=20&_sigthreshold=0.05&_msresflags=1025&_msresflags2=2&percolate=-1&percolate_rt=0) |
| 5 | [ALBU_RAT](http://www.matrixscience.com/cgi/protein_view.pl?file=../data/20110616/FtteInTet.dat&hit=1) | [ALBU_RAT](http://www.matrixscience.com/cgi/protein_view.pl?file=../data/20110613/FtteifawO.dat&hit=ALBU_RAT&db_idx=1&px=1&ave_thresh=23&_ignoreionsscorebelow=0&report=20&_sigthreshold=0.05&_msresflags=1025&_msresflags2=2&percolate=-1&percolate_rt=0) |
| 6 | [ALBU_RAT](http://www.matrixscience.com/cgi/protein_view.pl?file=../data/20110616/FtteInTem.dat&hit=1) | [ALBU_RAT](http://www.matrixscience.com/cgi/protein_view.pl?file=../data/20110602/FtterGsaE.dat&hit=ALBU_RAT&db_idx=1&px=1&ave_thresh=24&_ignoreionsscorebelow=0&report=20&_sigthreshold=0.05&_msresflags=1025&_msresflags2=2&percolate=-1&percolate_rt=0) |
| 7 | [ALBU_RAT](http://www.matrixscience.com/cgi/protein_view.pl?file=../data/20110616/FtteInTmT.dat&hit=1) | [ALBU_RAT](http://www.matrixscience.com/cgi/protein_view.pl?file=../data/20110602/FtterGsaE.dat&hit=ALBU_RAT&db_idx=1&px=1&ave_thresh=24&_ignoreionsscorebelow=0&report=20&_sigthreshold=0.05&_msresflags=1025&_msresflags2=2&percolate=-1&percolate_rt=0) |
| 8 | [ALBU_RAT](http://www.matrixscience.com/cgi/protein_view.pl?file=../data/20110616/FtteInTmO.dat&hit=1) | [ALBU_RAT](http://www.matrixscience.com/cgi/protein_view.pl?file=../data/20110613/FtteifawO.dat&hit=ALBU_RAT&db_idx=1&px=1&ave_thresh=23&_ignoreionsscorebelow=0&report=20&_sigthreshold=0.05&_msresflags=1025&_msresflags2=2&percolate=-1&percolate_rt=0) |
| 9 | [ANR16_RAT](http://www.matrixscience.com/cgi/protein_view.pl?file=../data/20110613/FtteiaYat.dat&hit=1) | NA |
| 10 | [ALBU_RAT](http://www.matrixscience.com/cgi/protein_view.pl?file=../data/20110613/FtteiaYne.dat&hit=1) | [ALBU_RAT](http://www.matrixscience.com/cgi/protein_view.pl?file=../data/20110613/FtteifuSR.dat&hit=ALBU_RAT&db_idx=1&px=1&ave_thresh=23&_ignoreionsscorebelow=0&report=20&_sigthreshold=0.05&_msresflags=1025&_msresflags2=2&percolate=-1&percolate_rt=0) |
| 11 | [ALBU_RAT](http://www.matrixscience.com/cgi/protein_view.pl?file=../data/20110616/FtteInTTE.dat&hit=1) | [ALBU_RAT](http://www.matrixscience.com/cgi/protein_view.pl?file=../data/20110613/FtteifuSR.dat&hit=ALBU_RAT&db_idx=1&px=1&ave_thresh=23&_ignoreionsscorebelow=0&report=20&_sigthreshold=0.05&_msresflags=1025&_msresflags2=2&percolate=-1&percolate_rt=0) |
| 12 | [PDIA3_RAT](http://www.matrixscience.com/cgi/protein_view.pl?file=../data/20110616/FtteInTTh.dat&hit=1) | PDIA4_RAT |
| 13 | [PDIA3_RAT](http://www.matrixscience.com/cgi/protein_view.pl?file=../data/20110616/FtteInTTR.dat&hit=1) | [PDIA3_RAT](http://www.matrixscience.com/cgi/protein_view.pl?file=../data/20110613/FtteifueO.dat&hit=PDIA3_RAT&db_idx=1&px=1&ave_thresh=22&_ignoreionsscorebelow=0&report=20&_sigthreshold=0.05&_msresflags=1025&_msresflags2=2&percolate=-1&percolate_rt=0) |
| 15 | [ACTB_RAT](http://www.matrixscience.com/cgi/protein_view.pl?file=../data/20110616/FtteInTOe.dat&hit=1) | [ACTB_RAT](http://www.matrixscience.com/cgi/protein_view.pl?file=../data/20110613/FtteifuTe.dat&hit=ACTB_RAT&db_idx=1&px=1&ave_thresh=19&_ignoreionsscorebelow=0&report=20&_sigthreshold=0.05&_msresflags=1025&_msresflags2=2&percolate=-1&percolate_rt=0) |
| 16 | [ACTG_RAT](http://www.matrixscience.com/cgi/protein_view.pl?file=../data/20110616/FtteInTwT.dat&hit=1) | NA |
| 17 | [ACTG_RAT](http://www.matrixscience.com/cgi/protein_view.pl?file=../data/20110616/FtteInTwS.dat&hit=1) | [ACTB_RAT](http://www.matrixscience.com/cgi/protein_view.pl?file=../data/20110602/FtterGeSh.dat&hit=ACTB_RAT&db_idx=1&px=1&ave_thresh=24&_ignoreionsscorebelow=0&report=20&_sigthreshold=0.05&_msresflags=1025&_msresflags2=2&percolate=-1&percolate_rt=0) |
| 18 | NA | [ACTB_RAT](http://www.matrixscience.com/cgi/protein_view.pl?file=../data/20110613/Ftteifuwe.dat&hit=ACTB_RAT&db_idx=1&px=1&ave_thresh=24&_ignoreionsscorebelow=0&report=20&_sigthreshold=0.05&_msresflags=1025&_msresflags2=2&percolate=-1&percolate_rt=0) |
| 19 | [ACTB_RAT](http://www.matrixscience.com/cgi/protein_view.pl?file=../data/20110616/FtteInTaT.dat&hit=1) | NA |
| 20 | [ACTB_RAT](http://www.matrixscience.com/cgi/protein_view.pl?file=../data/20110616/FtteInTaE.dat&hit=1) | NA |
| 21 | [ACTB_RAT](http://www.matrixscience.com/cgi/protein_view.pl?file=../data/20110616/FtteInTah.dat&hit=1) | NA |
| 22 | [K1C19_RAT](http://www.matrixscience.com/cgi/protein_view.pl?file=../data/20110616/FtteInTaR.dat&hit=1) | [K1C19_RAT](http://www.matrixscience.com/cgi/protein_view.pl?file=../data/20110602/FtterGeme.dat&hit=K1C19_RAT&db_idx=1&px=1&ave_thresh=24&_ignoreionsscorebelow=0&report=20&_sigthreshold=0.05&_msresflags=1025&_msresflags2=2&percolate=-1&percolate_rt=0) |
| 23 | [ATPB_RAT](http://www.matrixscience.com/cgi/protein_view.pl?file=../data/20110616/FtteInTtO.dat&hit=1) | [ATPB_RAT](http://www.matrixscience.com/cgi/protein_view.pl?file=../data/20110613/Ftteifunh.dat&hit=ATPB_RAT&db_idx=1&px=1&ave_thresh=23&_ignoreionsscorebelow=0&report=20&_sigthreshold=0.05&_msresflags=1025&_msresflags2=2&percolate=-1&percolate_rt=0) |
| 26 | [RSSA_RAT](http://www.matrixscience.com/cgi/protein_view.pl?file=../data/20110616/FtteInTth.dat&hit=1) | [RSSA_RAT](http://www.matrixscience.com/cgi/protein_view.pl?file=../data/20110602/FtterGTmT.dat&hit=RSSA_RAT&db_idx=1&px=1&ave_thresh=23&_ignoreionsscorebelow=0&report=20&_sigthreshold=0.05&_msresflags=1025&_msresflags2=2&percolate=-1&percolate_rt=0) |
| 27 | [RSSA_RAT](http://www.matrixscience.com/cgi/protein_view.pl?file=../data/20110616/FtteInTth.dat&hit=1) | [RSSA_RAT](http://www.matrixscience.com/cgi/protein_view.pl?file=../data/20110613/FtteifsSR.dat&hit=RSSA_RAT&db_idx=1&px=1&ave_thresh=22&_ignoreionsscorebelow=0&report=20&_sigthreshold=0.05&_msresflags=1025&_msresflags2=2&percolate=-1&percolate_rt=0) |
| 30 | [ACTB_RAT](http://www.matrixscience.com/cgi/protein_view.pl?file=../data/20110616/FtteInHOS.dat&hit=1) | [ACTB_RAT](http://www.matrixscience.com/cgi/protein_view.pl?file=../data/20110616/FtteInHOT.dat&hit=ACTB_RAT&db_idx=1&px=1&ave_thresh=24&_ignoreionsscorebelow=0&report=20&_sigthreshold=0.05&_msresflags=1025&_msresflags2=2&percolate=-1&percolate_rt=0) |
| 31 | NA | [K1C10_RAT](http://www.matrixscience.com/cgi/protein_view.pl?file=../data/20110616/FtteInHOR.dat&hit=K1C10_RAT&db_idx=1&px=1&ave_thresh=25&_ignoreionsscorebelow=0&report=20&_sigthreshold=0.05&_msresflags=1025&_msresflags2=2&percolate=-1&percolate_rt=0) |
| 32 | [TRUB1_RAT](http://www.matrixscience.com/cgi/protein_view.pl?file=../data/20110616/FtteInHaE.dat&hit=1) | NA |
| 33 | [RGN_RAT](http://www.matrixscience.com/cgi/protein_view.pl?file=../data/20110616/FtteInHaR.dat&hit=1) | [RGN_RAT](http://www.matrixscience.com/cgi/protein_view.pl?file=../data/20110602/FtterrcSm.dat&hit=RGN_RAT&db_idx=1&px=1&ave_thresh=24&_ignoreionsscorebelow=0&report=20&_sigthreshold=0.05&_msresflags=1025&_msresflags2=2&percolate=-1&percolate_rt=0) |
| 34 | [ANXA5_RAT](http://www.matrixscience.com/cgi/protein_view.pl?file=../data/20110601/FtteffuSL.dat&hit=1) | [ANXA5_RAT](http://www.matrixscience.com/cgi/protein_view.pl?file=../data/20110602/FtterrctE.dat&hit=ANXA5_RAT&db_idx=1&px=1&ave_thresh=25&_ignoreionsscorebelow=0&report=20&_sigthreshold=0.05&_msresflags=1025&_msresflags2=2&percolate=-1&percolate_rt=0) |
| 35 | [ANXA5_RAT](http://www.matrixscience.com/cgi/protein_view.pl?file=../data/20110616/FtteInHnm.dat&hit=1) | [ANXA5_RAT](http://www.matrixscience.com/cgi/protein_view.pl?file=../data/20110602/FtterrctE.dat&hit=ANXA5_RAT&db_idx=1&px=1&ave_thresh=25&_ignoreionsscorebelow=0&report=20&_sigthreshold=0.05&_msresflags=1025&_msresflags2=2&percolate=-1&percolate_rt=0) |
| 37 | NA | [1433Z_RAT](http://www.matrixscience.com/cgi/protein_view.pl?file=../data/20110616/FtteInEaR.dat&hit=1433Z_RAT&db_idx=1&px=1&ave_thresh=22&_ignoreionsscorebelow=0&report=20&_sigthreshold=0.05&_msresflags=1025&_msresflags2=2&percolate=-1&percolate_rt=0) |
| 38 | [1433B_RAT](http://www.matrixscience.com/cgi/protein_view.pl?file=../data/20110616/FtteInYeh.dat&hit=1) | [1433B_RAT](http://www.matrixscience.com/cgi/protein_view.pl?file=../data/20110616/FtteInYeT.dat&hit=1433B_RAT&db_idx=1&px=1&ave_thresh=24&_ignoreionsscorebelow=0&report=20&_sigthreshold=0.05&_msresflags=1025&_msresflags2=2&percolate=-1&percolate_rt=0) |
| 39 | [CPNS1_RAT](http://www.matrixscience.com/cgi/protein_view.pl?file=../data/20110616/FtteInYmS.dat&hit=1) | [CPNS1_RAT](http://www.matrixscience.com/cgi/protein_view.pl?file=../data/20110602/FtterrsST.dat&hit=CPNS1_RAT&db_idx=1&px=1&ave_thresh=23&_ignoreionsscorebelow=0&report=20&_sigthreshold=0.05&_msresflags=1025&_msresflags2=2&percolate=-1&percolate_rt=0) |
| 40 | [TRUB1_RAT](http://www.matrixscience.com/cgi/protein_view.pl?file=../data/20110616/FtteInYmR.dat&hit=1) | NA |
| 44 | [3HAO_RAT](http://www.matrixscience.com/cgi/protein_view.pl?file=../data/20110616/FtteInYwh.dat&hit=1) | [3HAO_RAT](http://www.matrixscience.com/cgi/protein_view.pl?file=../data/20110602/FtterreeL.dat&hit=3HAO_RAT&db_idx=1&px=1&ave_thresh=24&_ignoreionsscorebelow=0&report=20&_sigthreshold=0.05&_msresflags=1025&_msresflags2=2&percolate=-1&percolate_rt=0) |
| 47 | [HSPB1_RAT](http://www.matrixscience.com/cgi/protein_view.pl?file=../data/20110616/FtteInYat.dat&hit=1) | [HSPB1_RAT](http://www.matrixscience.com/cgi/protein_view.pl?file=../data/20110602/Ftterremm.dat&hit=HSPB1_RAT&db_idx=1&px=1&ave_thresh=25&_ignoreionsscorebelow=0&report=20&_sigthreshold=0.05&_msresflags=1025&_msresflags2=2&percolate=-1&percolate_rt=0) |
| 51 | NA | [MYL4_RAT](http://www.matrixscience.com/cgi/protein_view.pl?file=../data/20110602/FtterrHeh.dat&hit=MYL4_RAT&db_idx=1&px=1&ave_thresh=23&_ignoreionsscorebelow=0&report=20&_sigthreshold=0.05&_msresflags=1025&_msresflags2=2&percolate=-1&percolate_rt=0) |
| 52 | [HSPB1_RAT](http://www.matrixscience.com/cgi/protein_view.pl?file=../data/20110616/FtteInSaT.dat&hit=1) | [HSPB1_RAT](http://www.matrixscience.com/cgi/protein_view.pl?file=../data/20110602/Ftterremm.dat&hit=HSPB1_RAT&db_idx=1&px=1&ave_thresh=25&_ignoreionsscorebelow=0&report=20&_sigthreshold=0.05&_msresflags=1025&_msresflags2=2&percolate=-1&percolate_rt=0) |
| 61 | [ST1A1_RAT](http://www.matrixscience.com/cgi/protein_view.pl?file=../data/20110616/FtteIGcwe.dat&hit=1) | [ST1A1_RAT](http://www.matrixscience.com/cgi/protein_view.pl?file=../data/20110602/FtterrYTO.dat&hit=ST1A1_RAT&db_idx=1&px=1&ave_thresh=23&_ignoreionsscorebelow=0&report=20&_sigthreshold=0.05&_msresflags=1025&_msresflags2=2&percolate=-1&percolate_rt=0) |
| 62 | [SODC_RAT](http://www.matrixscience.com/cgi/protein_view.pl?file=../data/20110616/FtteIGcam.dat&hit=1) | [SODC_RAT](http://www.matrixscience.com/cgi/protein_view.pl?file=../data/20110602/FtterrYwO.dat&hit=SODC_RAT&db_idx=1&px=1&ave_thresh=24&_ignoreionsscorebelow=0&report=20&_sigthreshold=0.05&_msresflags=1025&_msresflags2=2&percolate=-1&percolate_rt=0) |
| 63 | [SODC_RAT](http://www.matrixscience.com/cgi/protein_view.pl?file=../data/20110616/FtteIGcam.dat&hit=1) | [SODC_RAT](http://www.matrixscience.com/cgi/protein_view.pl?file=../data/20110602/FtterrYwO.dat&hit=SODC_RAT&db_idx=1&px=1&ave_thresh=24&_ignoreionsscorebelow=0&report=20&_sigthreshold=0.05&_msresflags=1025&_msresflags2=2&percolate=-1&percolate_rt=0) |
| 69 | [CYB5_RAT](http://www.matrixscience.com/cgi/protein_view.pl?file=../data/20110616/FtteIGaST.dat&hit=1) | [CYB5_RAT](http://www.matrixscience.com/cgi/protein_view.pl?file=../data/20110602/FtterrSam.dat&hit=CYB5_RAT&db_idx=1&px=1&ave_thresh=24&_ignoreionsscorebelow=0&report=20&_sigthreshold=0.05&_msresflags=1025&_msresflags2=2&percolate=-1&percolate_rt=0) |
